# New incursions of H5N1 clade 2.3.4.4b highly pathogenic avian influenza viruses in wild birds, South Korea, October 2024

**DOI:** 10.3389/fvets.2024.1526118

**Published:** 2025-01-10

**Authors:** Young-Jae Si, Dong-Ju Kim, Sun-Hak Lee, Ye-Ram Seo, Hyesung Jeong, Suwoong Lee, Dong-Hun Lee

**Affiliations:** ^1^Wildlife Disease Research Team, National Institute of Wildlife Disease Control and Prevention, Gwangju, Republic of Korea; ^2^Avian Disease Laboratory, College of Veterinary Medicine, Konkuk University, Seoul, Republic of Korea; ^3^Wildlife Health Laboratory, College of Veterinary Medicine, Konkuk University, Seoul, Republic of Korea

**Keywords:** highly pathogenic avian influenza virus, H5N1, wild bird, Mandarin Duck, phylogenetic analysis

## 1 Introduction

Highly pathogenic avian influenza (HPAI) subtype H5Nx viruses of the A/Goose/Guangdong/1/1996 (Gs/Gd) lineage have led to substantial economic losses within the poultry industry and represent an ongoing public health threat (1). The Gs/Gd lineage H5 viruses not only have evolved into 10 primary clades 0–9 with their subclades but are also reassorted with other influenza A viruses (2–4). Notably, since 2020, clade 2.3.4.4b HPAI H5N1 viruses have caused outbreaks across a broad geographic range, including Asia, Europe, Africa, North America, South America, and Antarctica (5–7). The infections of HPAI H5N1 viruses in mammals including wild, domestic, and humans underscore the potential zoonotic risk and pandemic potential of these evolving H5 viruses (8).

In South Korea, the H5Nx clade 2.3.4.4b HPAI viruses caused multiple outbreaks. During October 2022–March 2023, a total of 16 different genotypes of H5N1 2.3.4.4b HPAIV, the Kor22-23A-P, were reported in wild birds, showing a high genetic diversity of clade 2.3.4.4b HPAIVs generated through frequent reassortment with other influenza A viruses (9). Between December 2023 and May 2024, H5N1 and H5N6 2.3.4.4b HPAI viruses were reported (10, 11), including 32 cases in poultry farms (home.kahis.go.kr) and 19 cases in wild birds (http://wadis.go.kr). No HPAI virus has been detected in South Korea since June 2024, despite large-scale active surveillance targeting both wild birds and poultry. In this study, we report the detection of H5N1 HPAI viruses isolated from a captured wild Mandarin duck (*Aix galericulata*) on 15 October 2024, and a Northern pintail (*Anas acuta*) found dead on 17 October 2024, during early-stage HPAI surveillance in fall migration of wild waterfowl into South Korea. To facilitate timely information sharing, we conducted genome sequencing of the H5N1 viruses using Illumina next-generation sequencing (NGS) technology and submitted the genome sequences to the GISAID database (https://www.gisaid.org). A comparative phylogenetic analysis was carried out to determine the virus's origin and genotype.

## 2 Materials and methods

### 2.1 Sample collection and virus isolation

On 15 October 2024, we captured eight wild Mandarin ducks along the Cheongmicheon in Gyeonggi-do Province, South Korea (GPS coordinate: 37°8′31.25″N, 127°22′52.23″E) as a part of the national wild bird surveillance program in South Korea (Supplementary Figure 1). On 17 October 2024, a dead Northern pintail was found at the Yongsu reservoir on Jeju Island (GPS coordinate: 33°30′18.28″N, 126°53′33.4″E). We collected oropharyngeal and cloacal swabs from the birds. Swab samples were placed in phosphate-buffered saline (PBS) containing 400 mg/ml of gentamicin and thoroughly homogenized by vortexing for 1 min. The supernatant of samples was filtered using a 0.45-μm Minisart Syringe Filter (Sartorius, Göttingen, Germany) after centrifugation of the sample at 3,000 rpm for 10 min and inoculated into 10-day-old specific-pathogen-free (SPF) embryonated chicken eggs. After 72 h of incubation at 37°C, the allantoic fluids were harvested and tested for hemagglutination activity (HA) using 10% chicken red blood cells. RNA was extracted from the hemagglutination-activity-positive allantoic fluid using the Maxwell RSC simply RNA Tissue Kit (Promega, Madison, WI, USA) according to the manufacturer's instructions and screened for the matrix (M) and H5 genes of the avian influenza virus using real-time reverse transcription-PCR (rRT-PCR) as previously described (12).

### 2.2 Whole-genome sequencing and sequence analysis

Complementary DNA was generated using the SuperScript III First-Strand Synthesis system (Invitrogen, Carlsbad, CA, USA), and the eight gene segments were amplified using AccuPrime Pfx DNA Polymerase (Invitrogen, Carlsbad, CA, USA) as previously described (13). DNA libraries were prepared using Nextera DNA Flex Library Prep Kit (Illumina, San Diego, CA, USA), which utilizes transposon-mediated tagmentation and adapter ligation, with dual-index barcodes according to the manufacturer's instructions. The complete genome was sequenced using the paired-end 150 Illumina MiSeq sequencing-by-synthesis platform. NGS raw reads were trimmed of adapters and low-quality bases using BBDuk version 38.84 by setting the minimum quality to 30 (14). Trimmed reads were assembled *de novo* using the SPAdes assembler 3.15.5. Trimmed reads were mapped to the top result from the GISAID EpiFlu database, identified from contigs, using Minimap 2.24 (https://github.com/lh3/minimap2) with default options and visualized on Geneious Prime software. The assembled genome sequences produced by reference-guided genome assembly were used to generate the final consensus genome sequences. The dataset presented in this study can be found in online repositories. The names of repositories and accession IDs are available through the GISAID(https://www.gisaid.org) EpiFlu database (accession ID: EPI_ISL_19528860 and EPI_ISL_19531393). H5 clade classification was performed using an online subspecies classification tool available in the BV-BRC (https://www.bvbrc.org/app/SubspeciesClassification). The consensus genome sequences were examined to identify molecular markers associated with mammalian host adaptation, pathogenicity, and drug resistance. We utilized the FluSurver mutation tool from the GISAID Initiative (15) and performed manual screening based on known markers impacting AIV biological properties (16). Identified amino acid substitutions in the HA segment are referenced according to H5 numbering.

### 2.3 Phylogenetic analysis

All eight consensus genome sequences were analyzed through the BLAST query function of the GISAID database (https://gisaid.org/). From the top 500 BLAST hits, identical sequences were filtered out using ElimDupes software (https://www.hiv.lanl.gov/content/sequence/elimdupesv2/elimdupes.html). Genome sequences were aligned using MAFFT software (17). Phylogenetic tree construction for each gene was conducted with RAxML v8.0 (18) using the general time reversible model for nucleotide substitution and the Gamma model for rate heterogeneity, with 1,000 bootstrap replicates. Interactive Tree of Life (iTOL) was used to visualize the tree of each gene (19). A cluster was regarded as distinct only when it had a bootstrap support value > 70 and a nucleotide sequence identity > 97%. The genotype G2b and G2d clade 2.3.4.4b H5N1 viruses identified from 2021 to 2022 (20, 21) were used to verify the genotypes of viruses. The A/goose/Hunan/SE284/2022(SE284) (H5N1) (2) was used to categorize genotype G2c viruses.

A Bayesian relaxed-clock phylogeny of the HA gene was reconstructed using BEAST version 1.10.4 (22), applying the Hasegawa, Kishino, and Yano substitution model with an uncorrelated log-normal distribution and a Gaussian Markov Random Field (GMRF) Bayesian skyride coalescent prior (23). The Markov Chain Monte Carlo (MCMC) process was run in parallel across three chains, each with 50 million iterations, and the results were combined after a 10% burn-in. All parameters achieved effective sample sizes >200 and were examined using TRACER v1.5 (http://tree.bio.ed.ac.uk/software/tracer/) (24). A maximum clade credibility (MCC) tree was created with TreeAnnotator and visualized using FigTree v1.4.4 (http://tree.bio.ed.ac.uk/software/figtree/). The time to the most recent common ancestor (tMRCA) was estimated based on the height values at common ancestor nodes.

## 3 Descriptive results

### 3.1 Isolation and genome sequencing of the virus

A live Mandarin duck out of eight captured on 15 October 2024, and a Northern pintail found dead on 17 October 2024 tested positive for influenza A virus via chicken embryo inoculation and rRT-PCR. We successfully isolated and sequenced the H5N1 HPAI viruses, designated as A/Mandarin duck/Korea/24WS005-2/H5N1/2024 (hereafter MD/24WS005-2) and A/Northern pintail/Korea/24WC025/H5N1/2024 (hereafter NP/24WC025). A total of 52,864 and 136,268 NGS reads were generated, respectively, resulting in complete coding genome sequences (CDSs) across all eight influenza virus segments and high average sequencing depth for each segment (>350). The viruses were identified as HPAI based on the presence of multiple basic amino acids at the HA proteolytic cleavage site (PLREKRRKR/G) (25) and classified as an H5 subtype clade 2.3.4.4b.

### 3.2 Genome analysis

**Figure 1:**
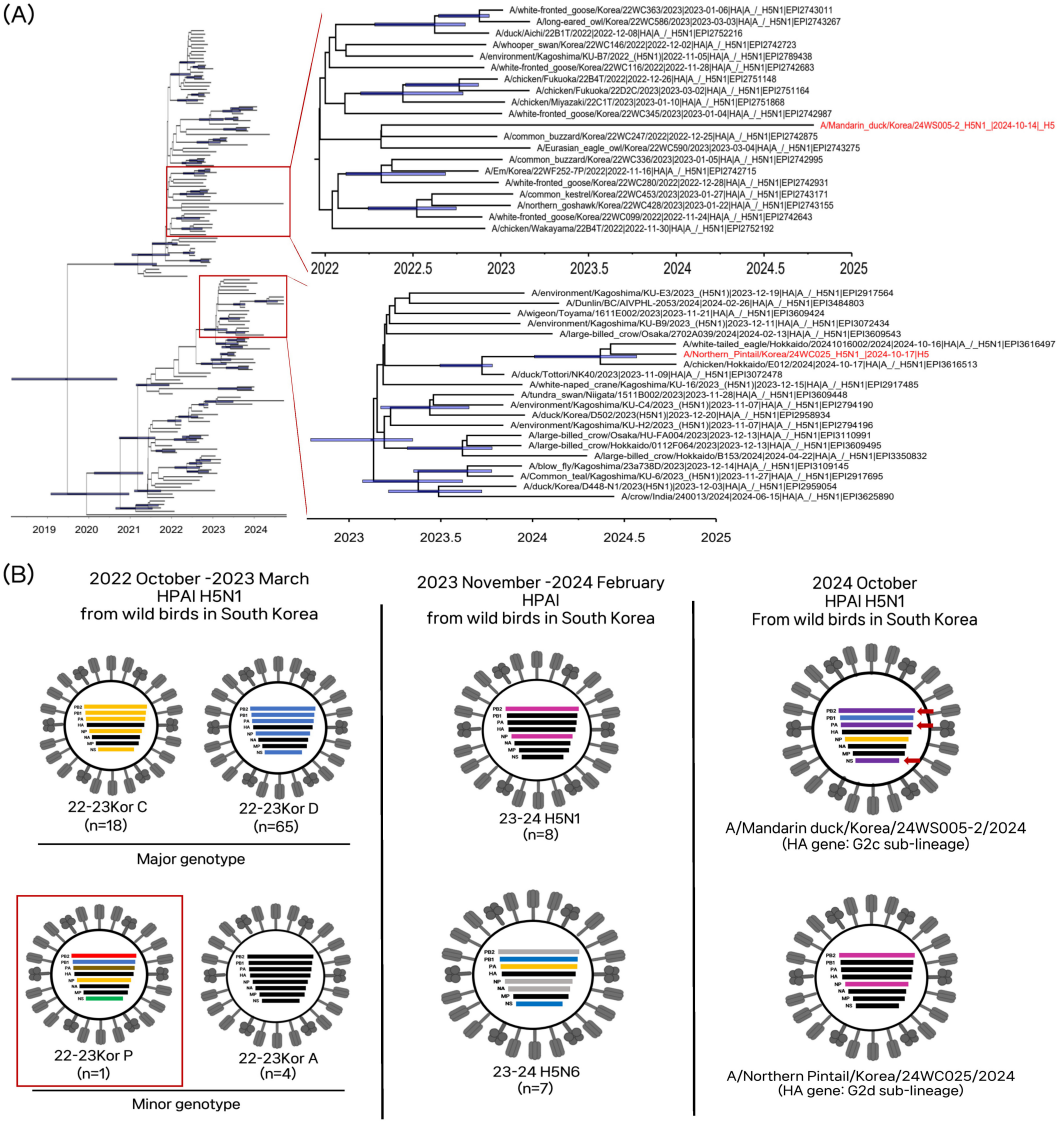
Phylogenetic analysis and genotypes of clade 2.3.4.4b H5N1 HPAI viruses found in wild birds in South Korea, October 2024. **(A)** Time-scaled Maximum clade credibility tree constructed using the hemagglutinin gene of clade 2.3.4.4 b H5N1 HPAI viruses. Taxa labeled in red indicate H5N1 isolates from South Korea, October 2024. Node bars represent 95% HPD of the node height with a posterior probability >0.5. The horizontal axis represents the decimal year. **(B)** Schematic representation of the origin of viruses isolates from South Korea, October 2024. Bars represent eight gene segments of the avian influenza virus in the following order (top to bottom): polymerase basic 2, polymerase basic 1, polymerase acidic, hemagglutinin, nucleoprotein, neuraminidase, matrix, and non-structural. Different bar colors indicate different virus origins estimated from maximum-likelihood phylogenetic trees. The red box indicates genotype P, detected in 2022–2023 in South Korea, which is genetically related to the A/Mandarin duck/Korea/24WS005-2/2024(H5N1) virus. The red arrows denote genes newly derived from low pathogenic viruses.

The NP/24WC025 virus and MD/24WS005-2 virus had different genome constellations, suggesting each virus had independently evolved and been introduced into South Korea (Figure 1). The genotype of the NP/24WC025 virus was identical to that of HPAI viruses circulating in Japan during 2023–2024. The HA gene belonged to the G2d sub-lineage (20, 21). The HA gene of NP/24WC025 virus shared a common ancestry with the clade 2.3.4.4b H5N1 HPAI viruses, A/white-tailed eagle/Hokkaido/2024, A/chicken/Hokkaido/E012/2024, which were concurrently identified in Japan. Their tMRCA was estimated to be 28 April 2024 (95% BCI: 10 January 2024–29 July 2024), suggesting that these H5N1 viruses are descendants of the G2d sub-lineage H5N1 viruses that circulated in Japan during early to mid-2024.

For the MD/24WS005-2 virus, the HA, NA, and M genes clustered with the H5Nx clade 2.3.4.4b HPAI viruses that mainly circulated in wild birds during 2022–2024 in Asia but did not form a well-supported monophyletic cluster with other viruses (Supplementary Figures 1D, F, G). The HA gene belonged to the G2c sub-lineage (2). In the Bayesian phylogenetic analysis of the HA gene, the MD/24WS005-2 virus clustered with clade 2.3.4.4b H5N6 HPAI viruses from wild birds in South Korea and Japan in the winter of 2023 but this grouping is not supported by posterior probability. All internal gene segments, except the M, clustered with LPAI viruses identified in the East-Asian Australasian migratory bird flyway, indicating the MD/24WS005-2 virus evolved via genome reassortment between the HA, NA, and M gene of clade 2.3.4.4b HPAI and the PB2, PB1, PA, NP, and NS of Eurasian LPAI viruses (Supplementary Figures 1A–C, E, H). Notably, the PB1, HA, NP, NA, and M genes shared a common ancestor with the A/eagle/Korea/22WC464/2023(H5N1) virus (Supplementary Figures 1B, D–G), which belonged to a minor genotype, the Kor22-23P, of H5N1 HPAI viruses that circulated in South Korea during the winter season of 2022–2023 (9). The PB2, PA, and NS genes are derived from the Eurasian LPAI gene pool circulating in wild bird populations (Supplementary Figures 1A, C, H). The high genetic diversity of avian influenza viruses in wild birds has contributed to the generation of multiple genotypes of clade 2.3.4.4b HPAI viruses as a donor gene pool of different genetic lineages (26). The long branch length and new genes derived from the LPAI gene pool suggest that it had circulated undetected for ~2 years and had undergone multiple reassortments with prevailing LPAI viruses in wild bird populations.

Genetic mutations associated with increased binding affinity to α-2,6 sialic acid receptors were found in the HA protein, including the N110S in MD/24WS005-2 virus, S154N in NP/24WC025 virus, and S133A and T156A in both viruses (Table 1). Both viruses carried the V588T mutation in the PB2 protein, known to enhance pathogenicity in mice. The K482R, associated with increased polymerase activity in mammalian cell lines, was identified in the NP/24WC025 virus. In the PB1 gene, mutations D3V and D622G, which are associated with enhanced polymerase activity and viral replication in avian and mammalian cell lines and increased virulence in mice, were present in both viruses. Mutations in the M protein (N30D, I43M, T215A, and P42S), known to increase pathogenicity in murine models, were identified in both viruses, along with the presence of the ESEV motif in the C-terminal of NS1 protein.

**Table 1:** Amino acid substitutions of A/Mandarin duck/Korea/24WS005-2/H5N1/2024 and A/Northern pintail/Korea/24WC025/H5N1/2024 associated with mammalian adaptations.

Gene^*^	**Mutations**	**Amino acid**		**Associated effect**
		**24WS005-2**	**24WC025**	
PB2	L89V	V	V	Increased virulence in mice
PB2	D256G	D	D	Increased polymerase activity in mammalian cell line
PB2	K482R	K	R	Increased polymerase activity in mammalian cell line
PB2	A588V	A	A	Increased virulence in mice
PB2	Q591K	Q	Q	Increased virulence in mice
PB2	V598T/I	T	T	Increased virulence in mice
PB2	E627K	E	E	Increased virulence in mice
PB2	D701N	D	D	Increased virulence in mice
PB1	D3V	V	V	Increased polymerase activity and viral replication in avian and mammalian cell lines
PB1	D622G	G	G	Increased polymerase activity and virulence in mice
HA	D94N	S	S	Increased binding affinity to α-2,6 sialic acid receptors
HA	N110S	S	P	Increased binding affinity to α-2,6 sialic acid receptors
HA	S123A	S	P	Increased binding affinity to α-2,6 sialic acid receptors
HA	S133A	A	A	Increased binding affinity to α-2,6 sialic acid receptors
HA	T139P	A	A	Increased binding affinity to α-2,6 sialic acid receptors
HA	S154N	D	N	Increased binding affinity to αα-2,6 sialic acid receptors
HA	T156A	A	A	Increased binding affinity to α-2,6 sialic acid receptors
HA	T188I	T	T	Increased binding affinity to α-2,6 sialic acid receptors
HA	V210I	V	V	Increased binding affinity to α-2,6 sialic acid receptors
HA	Q222L	Q	Q	Increased binding affinity to α-2,6 sialic acid receptors
HA	G224S	G	G	Increased binding affinity to α-2,6 sialic acid receptors
MP	N30D	D	D	Increased virulence in mice
MP	I43M	M	M	Increased virulence in mice
MP	T215A	A	A	Increased virulence in mice
NS1	P42S	S	S	Increased virulence in mice
NS1	80–84 DEL	TIASV	TITPV	Increased virulence in mice
NS1	ESEV	ESEV	ESEV	Increased virulence in mice

^*^Identified amino acid substitutions in the HA segment are referenced according to H5 numbering.

## 4 Conclusion

The clade 2.3.4.4b HPAI viruses have led to considerable economic losses in the poultry sector and pose a serious public health risk. In this study, we report the detection of clade 2.3.4.4b H5N1 HPAI viruses from wild birds during October 2024 in South Korea. Complete genome sequencing and analysis suggest that the MD/24WS005-2 virus and NP/24WC025 virus have persisted in the wild bird population in East Asia, independently evolved, and were introduced to South Korea during the 2024 fall migration of wild birds. Nationwide active surveillance of HPAI in wild birds facilitated the early detection of HPAI introductions in South Korea. The genome sequences of HPAI viruses shared rapidly via the GISAID database will serve as valuable reference data for future genomic surveillance and outbreak investigation of HPAI viruses.

## Data Availability

The datasets presented in this study can be found in online repositories. The names of the repository/repositories and accession number(s) can be found in the article/Supplementary material.

## Supplementary material

Supplementary Figure 1 The locations of H5N1 HPAI detection are marked on the map. The map data were generated using Google Earth (https://earth.google.com).

Supplementary Figure 2 Maximum-likelihood tree constructed using the complete coding nucleotide sequences of **(A)** polymerase basic protein 2, **(B)** polymerase basic protein 1, **(C)** polymerase acidic protein, **(D)** hemagglutinin protein, **(E)** nucleoprotein, **(F)** neuraminidase protein, **(G)** matrix protein, and **(H)** non-structural protein. Red taxa indicate the H5N1 isolates from South Korea, October 2024. Blue taxon indicates the A/eagle/Korea/22WC464/2023, a genotype P virus detected during 2022-2023 HPAI outbreak in South Korea. The background of HPAI isolates is shaded in blue. Numerical values at the nodes represent 1,000 bootstrap replicate value (%). Bootstrap value < 70 was removed from the tree.

Supplementary Figure 3 Time-scaled Maximum clade credibility tree constructed using the hemagglutinin gene of clade 2.3.4.4 b H5N1 HPAI viruses. Red indicates H5N1 isolates from South Korea, October 2024. Node bars represent 95% HPD of the node height with a posterior probability >0.5. The horizontal axis represents the decimal year.
