# Supplementary material for: New incursions of H5N1 clade 2.3.4.4b highly pathogenic avian influenza viruses in wild birds, South Korea, October 2024
Source: Front Vet Sci. 2025 Jan 10;11:1526118. doi: 10.3389/fvets.2024.1526118 (PMC11758627; doi:10.3389/fvets.2024.1526118)
Supplement: Supplementary Figure 1 — The locations of H5N1 HPAI detection are marked on the map. The map data were generated using Google Earth (https://earth.google.com). [file Data_Sheet_1.pdf]

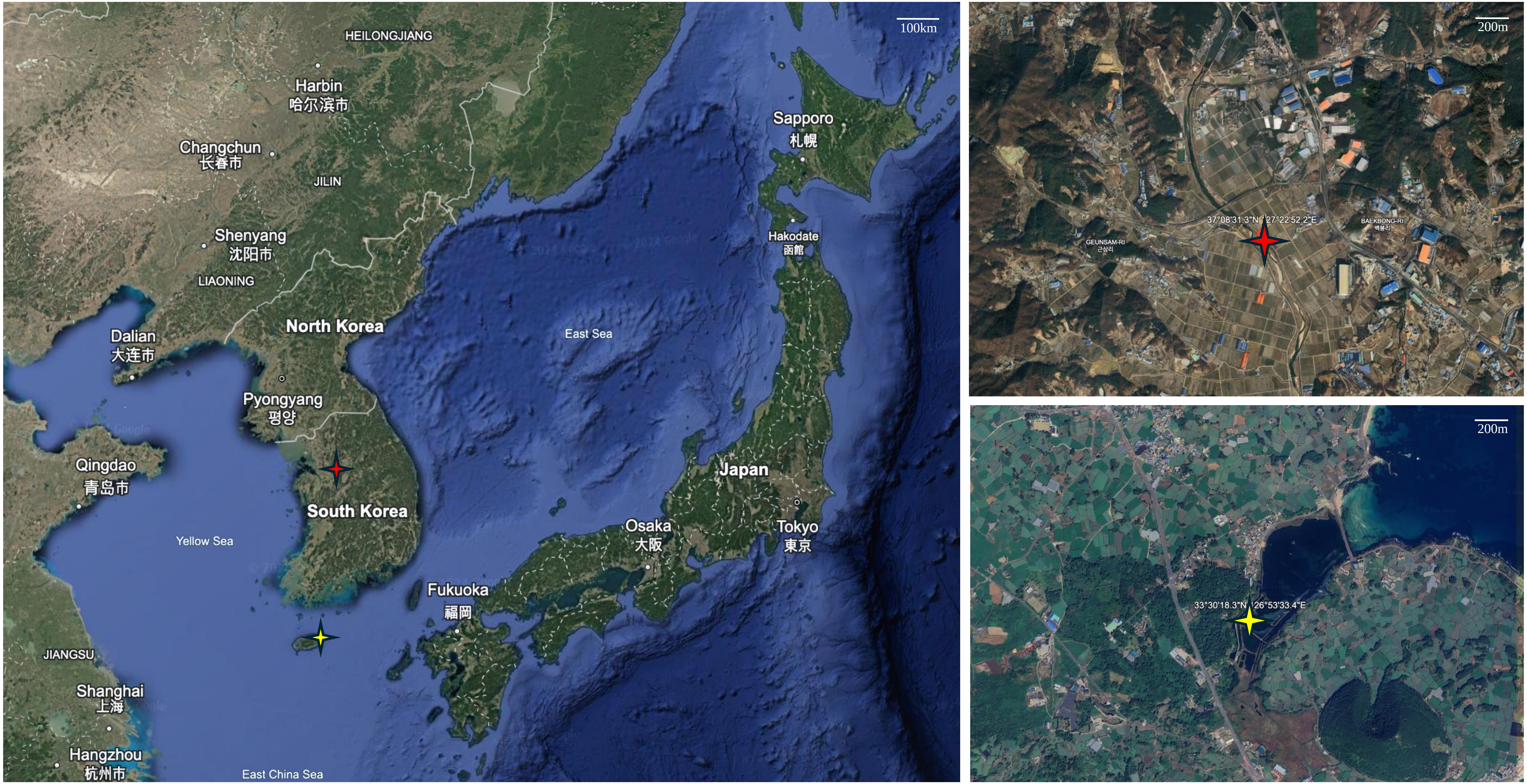

Cheongmicheon stream in Gyeonggi-do Province: A/Mandarin duck/Korea/24WS005-2/H5N1/2024(H5N1) found on 15 October 2024

Yongsu reservoir in Jeju island: A/Northern pintail/Korea/24WC025/H5N1/2024(H5N1) found on 17 October 2024
