# Supplementary material for: New incursions of H5N1 clade 2.3.4.4b highly pathogenic avian influenza viruses in wild birds, South Korea, October 2024
Source: Front Vet Sci. 2025 Jan 10;11:1526118. doi: 10.3389/fvets.2024.1526118 (PMC11758627; doi:10.3389/fvets.2024.1526118)
Supplement: Supplementary Figure 2 — Maximum-likelihood tree constructed using the complete coding nucleotide sequences of (A) polymerase basic protein 2, (B) polymerase basic protein 1, (C) polymerase acidic protein, (D) hemagglutinin protein, (E) nucleoprotein, (F) neuraminidase protein, (G) matrix protein, and (H) non-structural protein. Red taxa indicate the H5N1 isolates from South Korea, October 2024. Blue taxon indicates the A/eagle/Korea/22WC464/2023, a genotype P virus detected during 2022-2023 HPAI outbreak in South Korea. The background of HPAI isolates is shaded in blue. Numerical values at the nodes represent 1,000 bootstrap replicate value (%). Bootstrap value < 70 was removed from the tree. [file Data_Sheet_2.pdf]

PB2

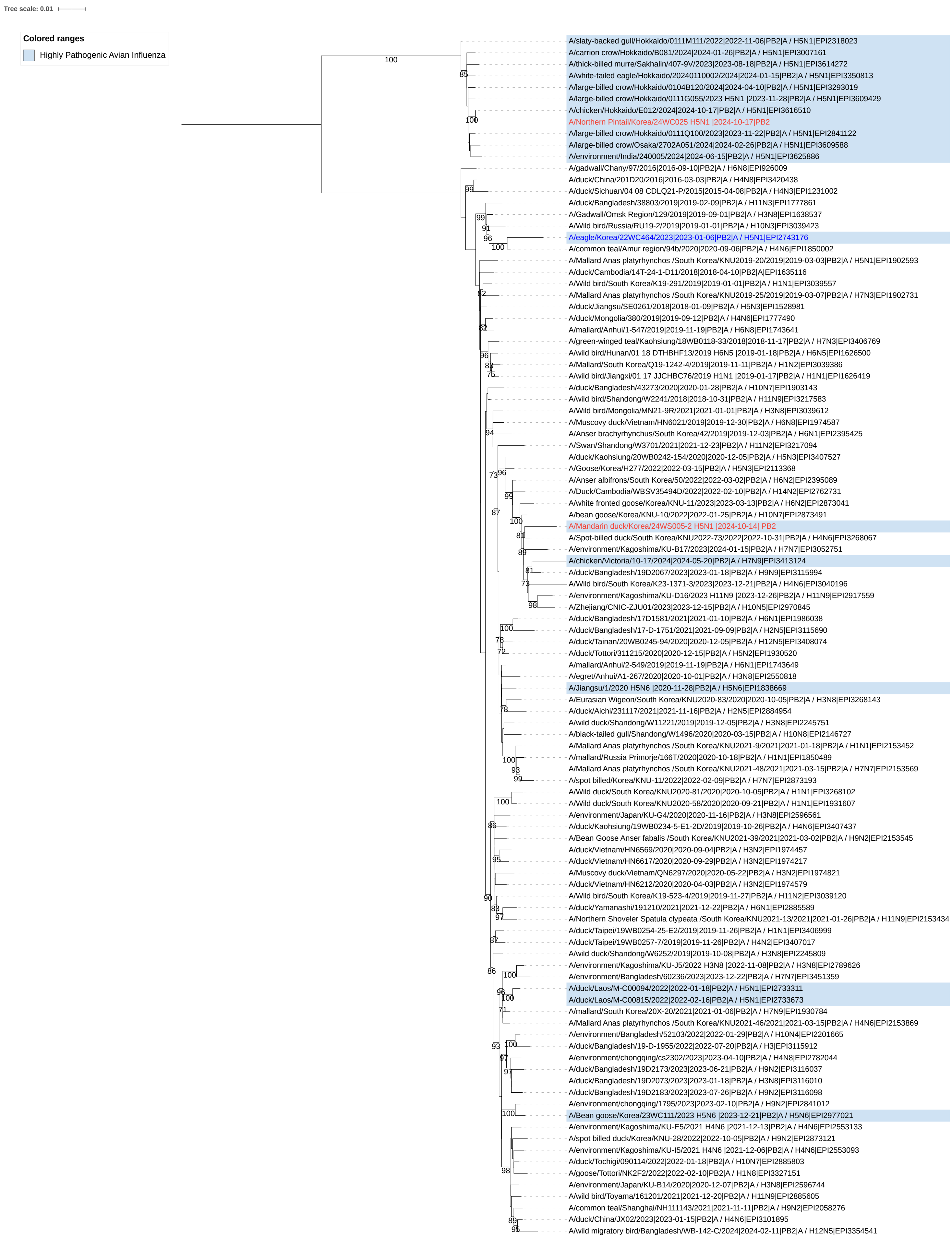

Tree scale: 0.01
Colored ranges
Highly Pathogenic Avian Influenza
A/slaty-backed gull/Hokkaido/0111M111/2022|2022-11-06|PB2|A / H5N1|EPI2318023
A/carrion crow/Hokkaido/B081/2024|2024-01-26|PB2|A / H5N1|EPI3007161
A/thick-billed murre/Sakhalin/407-9V/2023|2023-08-18|PB2|A / H5N1|EPI3614272
A/white-tailed eagle/Hokkaido/20240110002/2024|2024-01-15|PB2|A / H5N1|EPI3350813
A/large-billed crow/Hokkaido/0104B120/2024|2024-04-10|PB2|A / H5N1|EPI3293019
A/large-billed crow/Hokkaido/0111G055/2023 H5N1 |2023-11-28|PB2|A / H5N1|EPI3609429
A/chicken/Hokkaido/E012/2024|2024-10-17|PB2|A / H5N1|EPI3616510
A/Northern Pintail/Korea/24WC025 H5N1 |2024-10-17|PB2
A/large-billed crow/Hokkaido/0111Q100/2023|2023-11-22|PB2|A / H5N1|EPI2841122
A/large-billed crow/Osaka/2702A051/2024|2024-02-26|PB2|A / H5N1|EPI3609588
A/environment/India/240005/2024|2024-06-15|PB2|A / H5N1|EPI3625886
A/gadwall/Chany/97/2016|2016-09-10|PB2|A / H6N8|EPI926009
A/duck/China/201D20/2016|2016-03-03|PB2|A / H4N8|EPI3420438
A/duck/Sichuan/04 08 CDLQ21-P/2015|2015-04-08|PB2|A / H4N3|EPI1231002
A/duck/Bangladesh/38803/2019|2019-02-09|PB2|A / H11N3|EPI1777861
A/Gadwall/Omsk Region/129/2019|2019-09-01|PB2|A / H3N8|EPI1638537
A/Wild bird/Russia/RU19-2/2019|2019-01-01|PB2|A / H10N3|EPI3039423
A/eagle/Korea/22WC464/2023|2023-01-06|PB2|A / H5N1|EPI2743176
A/common teal/Amur region/94b/2020|2020-09-06|PB2|A / H4N6|EPI1850002
A/Mallard Anas platyrhynchos /South Korea/KNU2019-20/2019|2019-03-03|PB2|A / H5N1|EPI1902593
A/duck/Cambodia/14T-24-1-D11/2018|2018-04-10|PB2|A|EPI1635116
A/Wild bird/South Korea/K19-291/2019|2019-01-01|PB2|A / H1N1|EPI3039557
A/Mallard Anas platyrhynchos /South Korea/KNU2019-25/2019|2019-03-07|PB2|A / H7N3|EPI1902731
A/duck/Jiangsu/SE0261/2018|2018-01-09|PB2|A / H5N3|EPI1528981
A/duck/Mongolia/380/2019|2019-09-12|PB2|A / H4N6|EPI1777490
A/mallard/Anhui/1-547/2019|2019-11-19|PB2|A / H6N8|EPI1743641
A/green-winged teal/Kaohsiung/18WB0118-33/2018|2018-11-17|PB2|A / H7N3|EPI3406769
A/wild bird/Hunan/01 18 DTHBHF13/2019 H6N5 |2019-01-18|PB2|A / H6N5|EPI1626500
A/Mallard/South Korea/Q19-1242-4/2019|2019-11-11|PB2|A / H1N2|EPI3039386
A/wild bird/Jiangxi/01 17 JJCHBC76/2019 H1N1 |2019-01-17|PB2|A / H1N1|EPI1626419
A/duck/Bangladesh/43273/2020|2020-01-28|PB2|A / H10N7|EPI1903143
A/wild bird/Shandong/W2241/2018|2018-10-31|PB2|A / H11N9|EPI3217583
A/Wild bird/Mongolia/MN21-9R/2021|2021-01-01|PB2|A / H3N8|EPI3039612
A/Muscovy duck/Vietnam/HN6021/2019|2019-12-30|PB2|A / H6N8|EPI1974587
A/Anser brachyrhynchus/South Korea/42/2019|2019-12-03|PB2|A / H6N1|EPI2395425
A/Swan/Shandong/W3701/2021|2021-12-23|PB2|A / H11N2|EPI3217094
A/duck/Kaohsiung/20WB0242-154/2020|2020-12-05|PB2|A / H5N3|EPI3407527
A/Goose/Korea/H277/2022|2022-03-15|PB2|A / H5N3|EPI2113368
A/Anser albifrons/South Korea/50/2022|2022-03-02|PB2|A / H6N2|EPI2395089
A/Duck/Cambodia/WBSV35494D/2022|2022-02-10|PB2|A / H14N2|EPI2762731
A/white fronted goose/Korea/KNU-11/2023|2023-03-13|PB2|A / H6N2|EPI2873041
A/bean goose/Korea/KNU-10/2022|2022-01-25|PB2|A / H10N7|EPI2873491
A/Mandarin duck/Korea/24WS005-2 H5N1 |2024-10-14| PB2
A/Spot-billed duck/South Korea/KNU2022-73/2022|2022-10-31|PB2|A / H4N6|EPI3268067
A/environment/Kagoshima/KU-B17/2023|2024-01-15|PB2|A / H7N7|EPI3052751
A/chicken/Victoria/10-17/2024|2024-05-20|PB2|A / H7N9|EPI3413124
A/duck/Bangladesh/19D2067/2023|2023-01-18|PB2|A / H9N9|EPI3115994
A/Wild bird/South Korea/K23-1371-3/2023|2023-12-21|PB2|A / H4N6|EPI3040196
A/environment/Kagoshima/KU-D16/2023 H11N9 |2023-12-26|PB2|A / H11N9|EPI2917559
A/Zhejiang/CNIC-ZJU01/2023|2023-12-15|PB2|A / H10N5|EPI2970845
A/duck/Bangladesh/17D1581/2021|2021-01-10|PB2|A / H6N1|EPI1986038
A/duck/Bangladesh/17-D-1751/2021|2021-09-09|PB2|A / H2N5|EPI3115690
A/duck/Tainan/20WB0245-94/2020|2020-12-05|PB2|A / H12N5|EPI3408074
A/duck/Tottori/311215/2020|2020-12-15|PB2|A / H5N2|EPI1930520
A/mallard/Anhui/2-549/2019|2019-11-19|PB2|A / H6N1|EPI1743649
A/egret/Anhui/A1-267/2020|2020-10-01|PB2|A / H3N8|EPI2550818
A/Jiangsu/1/2020 H5N6 |2020-11-28|PB2|A / H5N6|EPI1838669
A/Eurasian Wigeon/South Korea/KNU2020-83/2020|2020-10-05|PB2|A / H3N8|EPI3268143
A/duck/Aichi/231117/2021|2021-11-16|PB2|A / H2N5|EPI2884954
A/wild duck/Shandong/W11221/2019|2019-12-05|PB2|A / H3N8|EPI2245751
A/black-tailed gull/Shandong/W1496/2020|2020-03-15|PB2|A / H10N8|EPI2146727
A/Mallard Anas platyrhynchos /South Korea/KNU2021-9/2021|2021-01-18|PB2|A / H1N1|EPI2153452
A/mallard/Russia Primorje/166T/2020|2020-10-18|PB2|A / H1N1|EPI1850489
A/Mallard Anas platyrhynchos /South Korea/KNU2021-48/2021|2021-03-15|PB2|A / H7N7|EPI2153569
A/spot billed/Korea/KNU-11/2022|2022-02-09|PB2|A / H7N7|EPI2873193
A/Wild duck/South Korea/KNU2020-81/2020|2020-10-05|PB2|A / H1N1|EPI3268102
A/Wild duck/South Korea/KNU2020-58/2020|2020-09-21|PB2|A / H1N1|EPI1931607
A/environment/Japan/KU-G4/2020|2020-11-16|PB2|A / H3N8|EPI2596561
A/duck/Kaohsiung/19WB0234-5-E1-2D/2019|2019-10-26|PB2|A / H4N6|EPI3407437
A/Bean Goose Anser fabalis /South Korea/KNU2021-39/2021|2021-03-02|PB2|A / H9N2|EPI2153545
A/duck/Vietnam/HN6569/2020|2020-09-04|PB2|A / H3N2|EPI1974457
A/duck/Vietnam/HN6617/2020|2020-09-29|PB2|A / H3N2|EPI1974217
A/Muscovy duck/Vietnam/QN6297/2020|2020-05-22|PB2|A / H3N2|EPI1974821
A/duck/Vietnam/HN6212/2020|2020-04-03|PB2|A / H3N2|EPI1974579
A/Wild bird/South Korea/K19-523-4/2019|2019-11-27|PB2|A / H11N2|EPI3039120
A/duck/Yamanashi/191210/2021|2021-12-22|PB2|A / H6N1|EPI2885589
A/Northern Shoveler Spatula clypeata /South Korea/KNU2021-13/2021|2021-01-26|PB2|A / H11N9|EPI2153434
A/duck/Taipei/19WB0254-25-E2/2019|2019-11-26|PB2|A / H1N1|EPI3406999
A/duck/Taipei/19WB0257-7/2019|2019-11-26|PB2|A / H4N2|EPI3407017
A/wild duck/Shandong/W6252/2019|2019-10-08|PB2|A / H3N8|EPI2245809
A/environment/Kagoshima/KU-J5/2022 H3N8 |2022-11-08|PB2|A / H3N8|EPI2789626
A/environment/Bangladesh/60236/2023|2023-12-22|PB2|A / H7N7|EPI3451359
A/duck/Laos/M-C00094/2022|2022-01-18|PB2|A / H5N1|EPI2733311
A/duck/Laos/M-C00815/2022|2022-02-16|PB2|A / H5N1|EPI2733673
A/mallard/South Korea/20X-20/2021|2021-01-06|PB2|A / H7N9|EPI1930784
A/Mallard Anas platyrhynchos /South Korea/KNU2021-46/2021|2021-03-15|PB2|A / H4N6|EPI2153869
A/environment/Bangladesh/52103/2022|2022-01-29|PB2|A / H10N4|EPI2201665
A/duck/Bangladesh/19-D-1955/2022|2022-07-20|PB2|A / H3|EPI3115912
A/environment/chongqing/cs2302/2023|2023-04-10|PB2|A / H4N8|EPI2782044
A/duck/Bangladesh/19D2173/2023|2023-06-21|PB2|A / H9N2|EPI3116037
A/duck/Bangladesh/19D2073/2023|2023-01-18|PB2|A / H3N8|EPI3116010
A/duck/Bangladesh/19D2183/2023|2023-07-26|PB2|A / H9N2|EPI3116098
A/environment/chongqing/1795/2023|2023-02-10|PB2|A / H9N2|EPI2841012
A/Bean goose/Korea/23WC111/2023 H5N6 |2023-12-21|PB2|A / H5N6|EPI2977021
A/environment/Kagoshima/KU-E5/2021 H4N6 |2021-12-13|PB2|A / H4N6|EPI2553133
A/spot billed duck/Korea/KNU-28/2022|2022-10-05|PB2|A / H9N2|EPI2873121
A/environment/Kagoshima/KU-I5/2021 H4N6 |2021-12-06|PB2|A / H4N6|EPI2553093
A/duck/Tochigi/090114/2022|2022-01-18|PB2|A / H10N7|EPI2885803
A/goose/Tottori/NK2F2/2022|2022-02-10|PB2|A / H1N8|EPI3327151
A/environment/Japan/KU-B14/2020|2020-12-07|PB2|A / H3N8|EPI2596744
A/wild bird/Toyama/161201/2021|2021-12-20|PB2|A / H11N9|EPI2885605
A/common teal/Shanghai/NH111143/2021|2021-11-11|PB2|A / H9N2|EPI2058276
A/duck/China/JX02/2023|2023-01-15|PB2|A / H4N6|EPI3101895
A/wild migratory bird/Bangladesh/WB-142-C/2024|2024-02-11|PB2|A / H12N5|EPI3354541

PB1

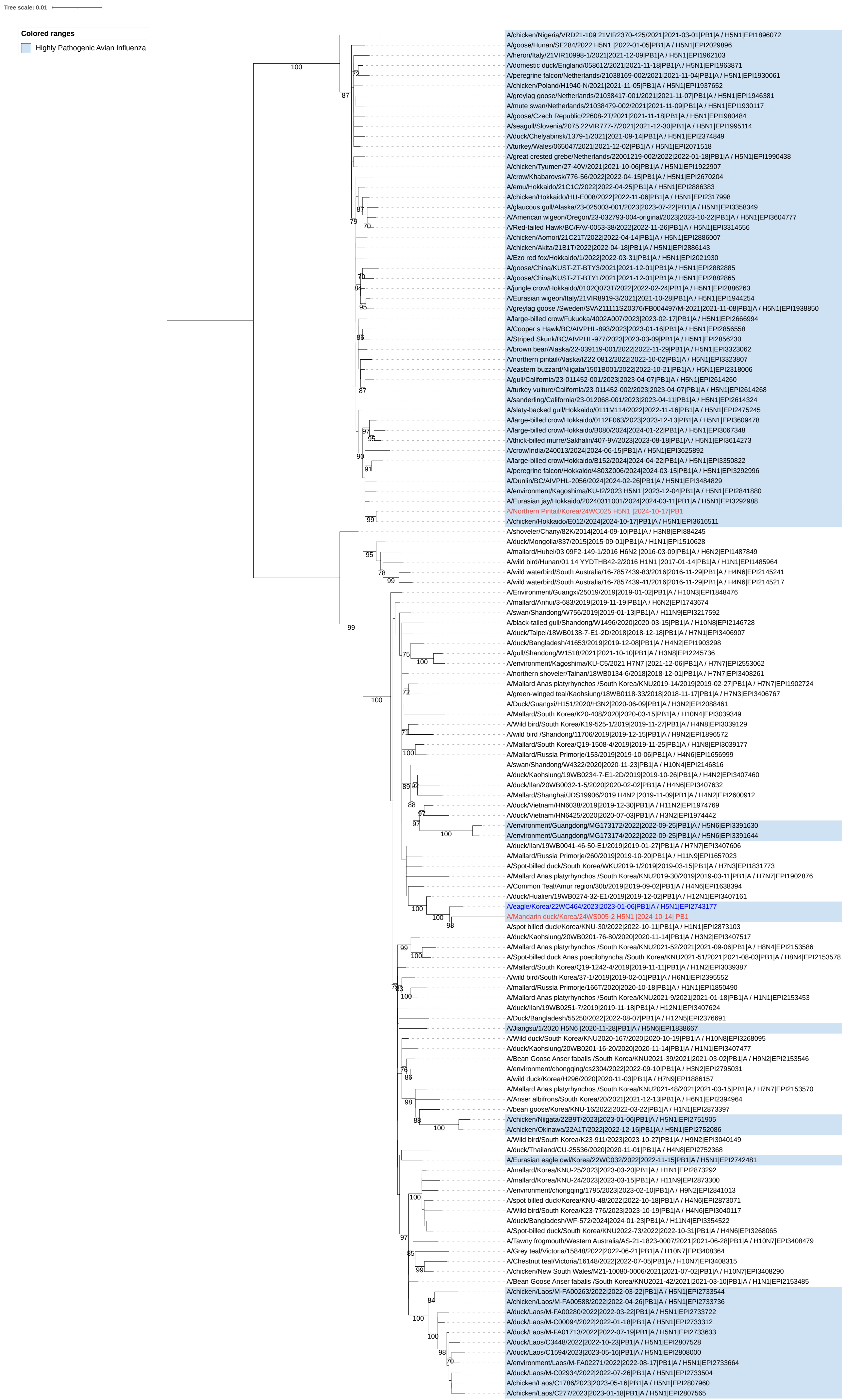

Tree scale: 0.01
Colored ranges
Highly Pathogenic Avian Influenza
A/chicken/Nigeria/VRD21-109 21VIR2370-425/2021|2021-03-01|PB1|A / H5N1|EPI1896072
A/goose/Hunan/SE284/2022 H5N1 |2022-01-05|PB1|A / H5N1|EPI2029896
A/heron/Italy/21VIR10998-1/2021|2021-12-09|PB1|A / H5N1|EPI1962103
A/domestic duck/England/058612/2021|2021-11-18|PB1|A / H5N1|EPI1963871
A/peregrine falcon/Netherlands/21038169-002/2021|2021-11-04|PB1|A / H5N1|EPI1930061
A/chicken/Poland/H1940-N/2021|2021-11-05|PB1|A / H5N1|EPI1937652
A/greylag goose/Netherlands/21038417-001/2021|2021-11-07|PB1|A / H5N1|EPI1946381
A/mute swan/Netherlands/21038479-002/2021|2021-11-09|PB1|A / H5N1|EPI1930117
A/goose/Czech Republic/22608-2T/2021|2021-11-18|PB1|A / H5N1|EPI1980484
A/seagull/Slovenia/2075 22VIR777-7/2021|2021-12-30|PB1|A / H5N1|EPI1995114
A/duck/Chelyabinsk/1379-1/2021|2021-09-14|PB1|A / H5N1|EPI2374849
A/turkey/Wales/065047/2021|2021-12-02|PB1|A / H5N1|EPI2071518
A/great crested grebe/Netherlands/22001219-002/2022|2022-01-18|PB1|A / H5N1|EPI1990438
A/chicken/Tyumen/27-40V/2021|2021-10-06|PB1|A / H5N1|EPI1922907
A/crow/Khabarovsk/776-56/2022|2022-04-15|PB1|A / H5N1|EPI2670204
A/emu/Hokkaido/21C1C/2022|2022-04-25|PB1|A / H5N1|EPI2886383
A/chicken/Hokkaido/HU-E008/2022|2022-11-06|PB1|A / H5N1|EPI2317998
A/glaucous gull/Alaska/23-025003-001/2023|2023-07-22|PB1|A / H5N1|EPI3358349
A/American wigeon/Oregon/23-032793-004-original/2023|2023-10-22|PB1|A / H5N1|EPI3604777
A/Red-tailed Hawk/BC/FAV-0053-38/2022|2022-11-26|PB1|A / H5N1|EPI3314556
A/chicken/Aomori/21C21T/2022|2022-04-14|PB1|A / H5N1|EPI2886007
A/chicken/Akita/21B1T/2022|2022-04-18|PB1|A / H5N1|EPI2886143
A/Ezo red fox/Hokkaido/1/2022|2022-03-31|PB1|A / H5N1|EPI2021930
A/goose/China/KUST-ZT-BTY3/2021|2021-12-01|PB1|A / H5N1|EPI2882885
A/goose/China/KUST-ZT-BTY1/2021|2021-12-01|PB1|A / H5N1|EPI2882865
A/jungle crow/Hokkaido/0102Q073T/2022|2022-02-24|PB1|A / H5N1|EPI2886263
A/Eurasian wigeon/Italy/21VIR8919-3/2021|2021-10-28|PB1|A / H5N1|EPI1944254
A/greylag goose /Sweden/SVA211111SZ0376/FB004497/M-2021|2021-11-08|PB1|A / H5N1|EPI1938850
A/large-billed crow/Fukuoka/4002A007/2023|2023-02-17|PB1|A / H5N1|EPI2666994
A/Cooper s Hawk/BC/AIVPHL-893/2023|2023-01-16|PB1|A / H5N1|EPI2856558
A/Striped Skunk/BC/AIVPHL-977/2023|2023-03-09|PB1|A / H5N1|EPI2856230
A/brown bear/Alaska/22-039119-001/2022|2022-11-29|PB1|A / H5N1|EPI3323062
A/northern pintail/Alaska/IZ22 0812/2022|2022-10-02|PB1|A / H5N1|EPI3323807
A/eastern buzzard/Niigata/1501B001/2022|2022-10-21|PB1|A / H5N1|EPI2318006
A/gull/California/23-011452-001/2023|2023-04-07|PB1|A / H5N1|EPI2614260
A/turkey vulture/California/23-011452-002/2023|2023-04-07|PB1|A / H5N1|EPI2614268
A/sanderling/California/23-012068-001/2023|2023-04-11|PB1|A / H5N1|EPI2614324
A/slaty-backed gull/Hokkaido/0111M114/2022|2022-11-16|PB1|A / H5N1|EPI2475245
A/large-billed crow/Hokkaido/0112F063/2023|2023-12-13|PB1|A / H5N1|EPI3609478
A/large-billed crow/Hokkaido/B080/2024|2024-01-22|PB1|A / H5N1|EPI3067348
A/thick-billed murre/Sakhalin/407-9V/2023|2023-08-18|PB1|A / H5N1|EPI3614273
A/crow/India/240013/2024|2024-06-15|PB1|A / H5N1|EPI3625892
A/large-billed crow/Hokkaido/B152/2024|2024-04-22|PB1|A / H5N1|EPI3350822
A/peregrine falcon/Hokkaido/4803Z006/2024|2024-03-15|PB1|A / H5N1|EPI3292996
A/Dunlin/BC/AIVPHL-2056/2024|2024-02-26|PB1|A / H5N1|EPI3484829
A/environment/Kagoshima/KU-I2/2023 H5N1 |2023-12-04|PB1|A / H5N1|EPI2841880
A/Eurasian jay/Hokkaido/20240311001/2024|2024-03-11|PB1|A / H5N1|EPI3292988
A/Northern Pintail/Korea/24WC025 H5N1 |2024-10-17|PB1
A/chicken/Hokkaido/E012/2024|2024-10-17|PB1|A / H5N1|EPI3616511
A/shoveler/Chany/82K/2014|2014-09-10|PB1|A / H3N8|EPI884245
A/duck/Mongolia/837/2015|2015-09-01|PB1|A / H1N1|EPI1510628
A/mallard/Hubei/03 09F2-149-1/2016 H6N2 |2016-03-09|PB1|A / H6N2|EPI1487849
A/wild bird/Hunan/01 14 YYDTHB42-2/2016 H1N1 |2017-01-14|PB1|A / H1N1|EPI1485964
A/wild waterbird/South Australia/16-7857439-83/2016|2016-11-29|PB1|A / H4N6|EPI2145241
A/wild waterbird/South Australia/16-7857439-41/2016|2016-11-29|PB1|A / H4N6|EPI2145217
A/Environment/Guangxi/25019/2019|2019-01-02|PB1|A / H10N3|EPI1848476
A/mallard/Anhui/3-683/2019|2019-11-19|PB1|A / H6N2|EPI1743674
A/swan/Shandong/W756/2019|2019-01-13|PB1|A / H11N9|EPI3217592
A/black-tailed gull/Shandong/W1496/2020|2020-03-15|PB1|A / H10N8|EPI2146728
A/duck/Taipei/18WB0138-7-E1-2D/2018|2018-12-18|PB1|A / H7N1|EPI3406907
A/duck/Bangladesh/41653/2019|2019-12-08|PB1|A / H4N2|EPI1903298
A/gull/Shandong/W1518/2021|2021-10-10|PB1|A / H3N8|EPI2245736
A/environment/Kagoshima/KU-C5/2021 H7N7 |2021-12-06|PB1|A / H7N7|EPI2553062
A/northern shoveler/Tainan/18WB0134-6/2018|2018-12-01|PB1|A / H7N7|EPI3408261
A/Mallard Anas platyrhynchos /South Korea/KNU2019-14/2019|2019-02-27|PB1|A / H7N7|EPI1902724
A/green-winged teal/Kaohsiung/18WB0118-33/2018|2018-11-17|PB1|A / H7N3|EPI3406767
A/Duck/Guangxi/H151/2020/H3N2|2020-06-09|PB1|A / H3N2|EPI2088461
A/Mallard/South Korea/K20-408/2020|2020-03-15|PB1|A / H10N4|EPI3039349
A/Wild bird/South Korea/K19-525-1/2019|2019-11-27|PB1|A / H4N8|EPI3039129
A/wild bird /Shandong/11706/2019|2019-12-15|PB1|A / H9N2|EPI1896572
A/Mallard/South Korea/Q19-1508-4/2019|2019-11-25|PB1|A / H1N8|EPI3039177
A/Mallard/Russia Primorje/153/2019|2019-10-06|PB1|A / H4N6|EPI1656999
A/swan/Shandong/W4322/2020|2020-11-23|PB1|A / H10N4|EPI2146816
A/duck/Kaohsiung/19WB0234-7-E1-2D/2019|2019-10-26|PB1|A / H4N2|EPI3407460
A/duck/Ilan/20WB0032-1-5/2020|2020-02-02|PB1|A / H4N6|EPI3407632
A/Mallard/Shanghai/JDS19906/2019 H4N2 |2019-11-09|PB1|A / H4N2|EPI2600912
A/duck/Vietnam/HN6038/2019|2019-12-30|PB1|A / H11N2|EPI1974769
A/duck/Vietnam/HN6425/2020|2020-07-03|PB1|A / H3N2|EPI1974442
A/environment/Guangdong/MG173172/2022|2022-09-25|PB1|A / H5N6|EPI3391630
A/environment/Guangdong/MG173174/2022|2022-09-25|PB1|A / H5N6|EPI3391644
A/duck/Ilan/19WB0041-46-50-E1/2019|2019-01-27|PB1|A / H7N7|EPI3407606
A/Mallard/Russia Primorje/260/2019|2019-10-20|PB1|A / H11N9|EPI1657023
A/Spot-billed duck/South Korea/WKU2019-1/2019|2019-03-15|PB1|A / H7N3|EPI1831773
A/Mallard Anas platyrhynchos /South Korea/KNU2019-30/2019|2019-03-11|PB1|A / H7N7|EPI1902876
A/Common Teal/Amur region/30b/2019|2019-09-02|PB1|A / H4N6|EPI1638394
A/duck/Hualien/19WB0274-32-E1/2019|2019-12-02|PB1|A / H12N1|EPI3407161
A/eagle/Korea/22WC464/2023|2023-01-06|PB1|A / H5N1|EPI2743177
A/Mandarin duck/Korea/24WS005-2 H5N1 |2024-10-14| PB1
A/spot billed duck/Korea/KNU-30/2022|2022-10-11|PB1|A / H1N1|EPI2873103
A/duck/Kaohsiung/20WB0201-76-80/2020|2020-11-14|PB1|A / H3N2|EPI3407517
A/Mallard Anas platyrhynchos /South Korea/KNU2021-52/2021|2021-09-06|PB1|A / H8N4|EPI2153586
A/Spot-billed duck Anas poecilohyncha /South Korea/KNU2021-51/2021|2021-08-03|PB1|A / H8N4|EPI2153578
A/Mallard/South Korea/Q19-1242-4/2019|2019-11-11|PB1|A / H1N2|EPI3039387
A/wild bird/South Korea/37-1/2019|2019-02-01|PB1|A / H6N1|EPI2395552
A/mallard/Russia Primorje/166T/2020|2020-10-18|PB1|A / H1N1|EPI1850490
A/Mallard Anas platyrhynchos /South Korea/KNU2021-9/2021|2021-01-18|PB1|A / H1N1|EPI2153453
A/duck/Ilan/19WB0251-7/2019|2019-11-18|PB1|A / H12N1|EPI3407624
A/Duck/Bangladesh/55250/2022|2022-08-07|PB1|A / H12N5|EPI2376691
A/Jiangsu/1/2020 H5N6 |2020-11-28|PB1|A / H5N6|EPI1838667
A/Wild duck/South Korea/KNU2020-167/2020|2020-10-19|PB1|A / H10N8|EPI3268095
A/duck/Kaohsiung/20WB0201-16-20/2020|2020-11-14|PB1|A / H1N1|EPI3407477
A/Bean Goose Anser fabalis /South Korea/KNU2021-39/2021|2021-03-02|PB1|A / H9N2|EPI2153546
A/environment/chongqing/cs2304/2022|2022-09-10|PB1|A / H3N2|EPI2795031
A/wild duck/Korea/H296/2020|2020-11-03|PB1|A / H7N9|EPI1886157
A/Mallard Anas platyrhynchos /South Korea/KNU2021-48/2021|2021-03-15|PB1|A / H7N7|EPI2153570
A/Anser albifrons/South Korea/20/2021|2021-12-13|PB1|A / H6N1|EPI2394964
A/bean goose/Korea/KNU-16/2022|2022-03-22|PB1|A / H1N1|EPI2873397
A/chicken/Niigata/22B9T/2023|2023-01-06|PB1|A / H5N1|EPI2751905
A/chicken/Okinawa/22A1T/2022|2022-12-16|PB1|A / H5N1|EPI2752086
A/Wild bird/South Korea/K23-911/2023|2023-10-27|PB1|A / H9N2|EPI3040149
A/duck/Thailand/CU-25536/2020|2020-11-01|PB1|A / H4N8|EPI2752368
A/Eurasian eagle owl/Korea/22WC032/2022|2022-11-15|PB1|A / H5N1|EPI2742481
A/mallard/Korea/KNU-25/2023|2023-03-20|PB1|A / H1N1|EPI2873292
A/mallard/Korea/KNU-24/2023|2023-03-15|PB1|A / H11N9|EPI2873300
A/environment/chongqing/1795/2023|2023-02-10|PB1|A / H9N2|EPI2841013
A/spot billed duck/Korea/KNU-48/2022|2022-10-18|PB1|A / H4N6|EPI2873071
A/Wild bird/South Korea/K23-776/2023|2023-10-19|PB1|A / H4N6|EPI3040117
A/duck/Bangladesh/WF-572/2024|2024-01-23|PB1|A / H11N4|EPI3354522
A/Spot-billed duck/South Korea/KNU2022-73/2022|2022-10-31|PB1|A / H4N6|EPI3268065
A/Tawny frogmouth/Western Australia/AS-21-1823-0007/2021|2021-06-28|PB1|A / H10N7|EPI3408479
A/Grey teal/Victoria/15848/2022|2022-06-21|PB1|A / H10N7|EPI3408364
A/Chestnut teal/Victoria/16148/2022|2022-07-05|PB1|A / H10N7|EPI3408315
A/chicken/New South Wales/M21-10080-0006/2021|2021-07-02|PB1|A / H10N7|EPI3408290
A/Bean Goose Anser fabalis /South Korea/KNU2021-42/2021|2021-03-10|PB1|A / H1N1|EPI2153485
A/chicken/Laos/M-FA00263/2022|2022-03-22|PB1|A / H5N1|EPI2733544
A/chicken/Laos/M-FA00588/2022|2022-04-26|PB1|A / H5N1|EPI2733736
A/duck/Laos/M-FA00280/2022|2022-03-22|PB1|A / H5N1|EPI2733722
A/duck/Laos/M-C00094/2022|2022-01-18|PB1|A / H5N1|EPI2733312
A/duck/Laos/M-FA01713/2022|2022-07-19|PB1|A / H5N1|EPI2733633
A/duck/Laos/C3448/2022|2022-10-23|PB1|A / H5N1|EPI2807528
A/duck/Laos/C1594/2023|2023-05-16|PB1|A / H5N1|EPI2808000
A/environment/Laos/M-FA02271/2022|2022-08-17|PB1|A / H5N1|EPI2733664
A/duck/Laos/M-C02934/2022|2022-07-26|PB1|A / H5N1|EPI2733504
A/chicken/Laos/C1786/2023|2023-05-16|PB1|A / H5N1|EPI2807960
A/chicken/Laos/C277/2023|2023-01-18|PB1|A / H5N1|EPI2807565

PA

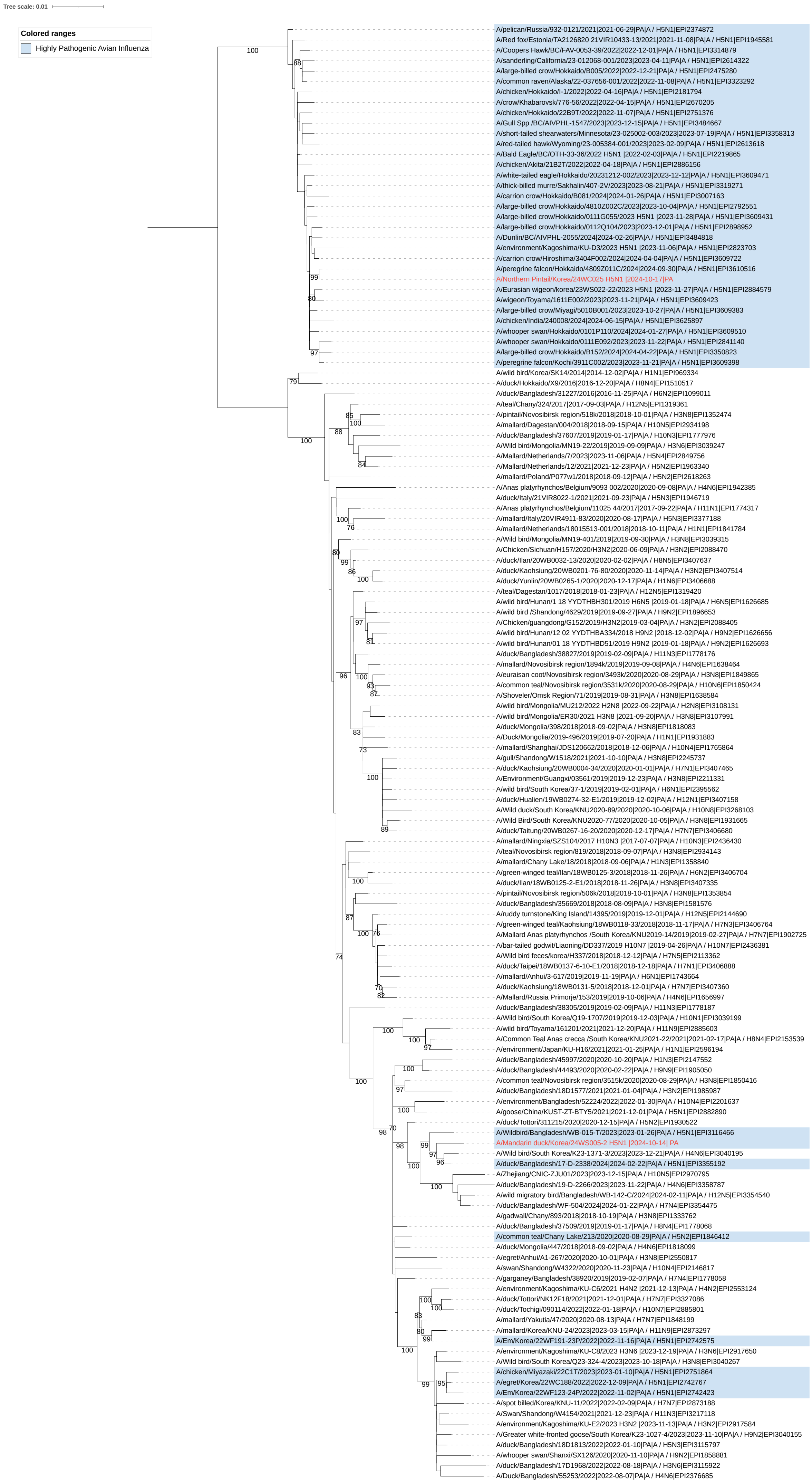
Tree scale: 0.01
Colored ranges
Highly Pathogenic Avian Influenza
A/pelican/Russia/932-0121/2021|2021-06-29|PA|A / H5N1|EPI2374872
A/Red fox/Estonia/TA2126820 21VIR10433-13/2021|2021-11-08|PA|A / H5N1|EPI1945581
A/Coopers Hawk/BC/FAV-0053-39/2022|2022-12-01|PA|A / H5N1|EPI3314879
A/sanderling/California/23-012068-001/2023|2023-04-11|PA|A / H5N1|EPI2614322
A/large-billed crow/Hokkaido/B005/2022|2022-12-21|PA|A / H5N1|EPI2475280
A/common raven/Alaska/22-037656-001/2022|2022-11-08|PA|A / H5N1|EPI3323292
A/chicken/Hokkaido/I-1/2022|2022-04-16|PA|A / H5N1|EPI2181794
A/crow/Khabarovsk/776-56/2022|2022-04-15|PA|A / H5N1|EPI2670205
A/chicken/Hokkaido/22B9T/2022|2022-11-07|PA|A / H5N1|EPI2751376
A/Gull Spp /BC/AIVPHL-1547/2023|2023-12-15|PA|A / H5N1|EPI3484667
A/short-tailed shearwaters/Minnesota/23-025002-003/2023|2023-07-19|PA|A / H5N1|EPI3358313
A/red-tailed hawk/Wyoming/23-005384-001/2023|2023-02-09|PA|A / H5N1|EPI2613618
A/Bald Eagle/BC/OTH-33-36/2022 H5N1 |2022-02-03|PA|A / H5N1|EPI2219865
A/chicken/Akita/21B2T/2022|2022-04-18|PA|A / H5N1|EPI2886156
A/white-tailed eagle/Hokkaido/20231212-002/2023|2023-12-12|PA|A / H5N1|EPI3609471
A/thick-billed murre/Sakhalin/407-2V/2023|2023-08-21|PA|A / H5N1|EPI3319271
A/carrion crow/Hokkaido/B081/2024|2024-01-26|PA|A / H5N1|EPI3007163
A/large-billed crow/Hokkaido/4810Z002C/2023|2023-10-04|PA|A / H5N1|EPI2792551
A/large-billed crow/Hokkaido/0111G055/2023 H5N1 |2023-11-28|PA|A / H5N1|EPI3609431
A/large-billed crow/Hokkaido/0112Q104/2023|2023-12-01|PA|A / H5N1|EPI2898952
A/Dunlin/BC/AIVPHL-2055/2024|2024-02-26|PA|A / H5N1|EPI3484818
A/environment/Kagoshima/KU-D3/2023 H5N1 |2023-11-06|PA|A / H5N1|EPI2823703
A/carrion crow/Hiroshima/3404F002/2024|2024-04-04|PA|A / H5N1|EPI3609722
A/peregrine falcon/Hokkaido/4809Z011C/2024|2024-09-30|PA|A / H5N1|EPI3610516
A/Northern Pintail/Korea/24WC025 H5N1 |2024-10-17|PA
A/Eurasian wigeon/korea/23WS022-22/2023 H5N1 |2023-11-27|PA|A / H5N1|EPI2884579
A/wigeon/Toyama/1611E002/2023|2023-11-21|PA|A / H5N1|EPI3609423
A/large-billed crow/Miyagi/5010B001/2023|2023-10-27|PA|A / H5N1|EPI3609383
A/chicken/India/240008/2024|2024-06-15|PA|A / H5N1|EPI3625897
A/whooper swan/Hokkaido/0101P110/2024|2024-01-27|PA|A / H5N1|EPI3609510
A/whooper swan/Hokkaido/0111E092/2023|2023-11-22|PA|A / H5N1|EPI2841140
A/large-billed crow/Hokkaido/B152/2024|2024-04-22|PA|A / H5N1|EPI3350823
A/peregrine falcon/Kochi/3911C002/2023|2023-11-21|PA|A / H5N1|EPI3609398
A/wild bird/Korea/SK14/2014|2014-12-02|PA|A / H1N1|EPI969334
A/duck/Hokkaido/X9/2016|2016-12-20|PA|A / H8N4|EPI1510517
A/duck/Bangladesh/31227/2016|2016-11-25|PA|A / H6N2|EPI1099011
A/teal/Chany/324/2017|2017-09-03|PA|A / H12N5|EPI1319361
A/pintail/Novosibirsk region/518k/2018|2018-10-01|PA|A / H3N8|EPI1352474
A/mallard/Dagestan/004/2018|2018-09-15|PA|A / H10N5|EPI2934198
A/duck/Bangladesh/37607/2019|2019-01-17|PA|A / H10N3|EPI1777976
A/Wild bird/Mongolia/MN19-22/2019|2019-09-09|PA|A / H3N6|EPI3039247
A/Mallard/Netherlands/7/2023|2023-11-06|PA|A / H5N4|EPI2849756
A/Mallard/Netherlands/12/2021|2021-12-23|PA|A / H5N2|EPI1963340
A/mallard/Poland/P077w1/2018|2018-09-12|PA|A / H5N2|EPI2618263
A/Anas platyrhynchos/Belgium/9093 002/2020|2020-09-08|PA|A / H4N6|EPI1942385
A/duck/Italy/21VIR8022-1/2021|2021-09-23|PA|A / H5N3|EPI1946719
A/Anas platyrhynchos/Belgium/11025 44/2017|2017-09-22|PA|A / H11N1|EPI1774317
A/mallard/Italy/20VIR4911-83/2020|2020-08-17|PA|A / H5N3|EPI3377188
A/mallard/Netherlands/18015513-001/2018|2018-10-11|PA|A / H1N1|EPI1841784
A/Wild bird/Mongolia/MN19-401/2019|2019-09-30|PA|A / H3N8|EPI3039315
A/Chicken/Sichuan/H157/2020/H3N2|2020-06-09|PA|A / H3N2|EPI2088470
A/duck/Ilan/20WB0032-13/2020|2020-02-02|PA|A / H8N5|EPI3407637
A/duck/Kaohsiung/20WB0201-76-80/2020|2020-11-14|PA|A / H3N2|EPI3407514
A/duck/Yunlin/20WB0265-1/2020|2020-12-17|PA|A / H1N6|EPI3406688
A/teal/Dagestan/1017/2018|2018-01-23|PA|A / H12N5|EPI1319420
A/wild bird/Hunan/1 18 YYDTHBH301/2019 H6N5 |2019-01-18|PA|A / H6N5|EPI1626685
A/wild bird /Shandong/4629/2019|2019-09-27|PA|A / H9N2|EPI1896653
A/Chicken/guangdong/G152/2019/H3N2|2019-03-04|PA|A / H3N2|EPI2088405
A/wild bird/Hunan/12 02 YYDTHBA334/2018 H9N2 |2018-12-02|PA|A / H9N2|EPI1626656
A/wild bird/Hunan/01 18 YYDTHBD51/2019 H9N2 |2019-01-18|PA|A / H9N2|EPI1626693
A/duck/Bangladesh/38827/2019|2019-02-09|PA|A / H11N3|EPI1778176
A/mallard/Novosibirsk region/1894k/2019|2019-09-08|PA|A / H4N6|EPI1638464
A/euraisan coot/Novosibirsk region/3493k/2020|2020-08-29|PA|A / H3N8|EPI1849865
A/common teal/Novosibirsk region/3531k/2020|2020-08-29|PA|A / H10N6|EPI1850424
A/Shoveler/Omsk Region/71/2019|2019-08-31|PA|A / H3N8|EPI1638584
A/wild bird/Mongolia/MU212/2022 H2N8 |2022-09-22|PA|A / H2N8|EPI3108131
A/wild bird/Mongolia/ER30/2021 H3N8 |2021-09-20|PA|A / H3N8|EPI3107991
A/duck/Mongolia/398/2018|2018-09-02|PA|A / H3N8|EPI1818083
A/Duck/Mongolia/2019-496/2019|2019-07-20|PA|A / H1N1|EPI1931883
A/mallard/Shanghai/JDS120662/2018|2018-12-06|PA|A / H10N4|EPI1765864
A/gull/Shandong/W1518/2021|2021-10-10|PA|A / H3N8|EPI2245737
A/duck/Kaohsiung/20WB0004-34/2020|2020-01-01|PA|A / H7N1|EPI3407465
A/Environment/Guangxi/03561/2019|2019-12-23|PA|A / H3N8|EPI2211331
A/wild bird/South Korea/37-1/2019|2019-02-01|PA|A / H6N1|EPI2395562
A/duck/Hualien/19WB0274-32-E1/2019|2019-12-02|PA|A / H12N1|EPI3407158
A/Wild duck/South Korea/KNU2020-89/2020|2020-10-06|PA|A / H10N8|EPI3268103
A/Wild Bird/South Korea/KNU2020-77/2020|2020-10-05|PA|A / H3N8|EPI1931665
A/duck/Taitung/20WB0267-16-20/2020|2020-12-17|PA|A / H7N7|EPI3406680
A/mallard/Ningxia/SZS104/2017 H10N3 |2017-07-07|PA|A / H10N3|EPI2436430
A/teal/Novosibirsk region/819/2018|2018-09-07|PA|A / H3N8|EPI2934143
A/mallard/Chany Lake/18/2018|2018-09-06|PA|A / H1N3|EPI1358840
A/green-winged teal/Ilan/18WB0125-3/2018|2018-11-26|PA|A / H6N2|EPI3406704
A/duck/Ilan/18WB0125-2-E1/2018|2018-11-26|PA|A / H3N8|EPI3407335
A/pintail/Novosibirsk region/506k/2018|2018-10-01|PA|A / H3N8|EPI1353854
A/duck/Bangladesh/35669/2018|2018-08-09|PA|A / H3N8|EPI1581576
A/ruddy turnstone/King Island/14395/2019|2019-12-01|PA|A / H12N5|EPI2144690
A/green-winged teal/Kaohsiung/18WB0118-33/2018|2018-11-17|PA|A / H7N3|EPI3406764
A/Mallard Anas platyrhynchos /South Korea/KNU2019-14/2019|2019-02-27|PA|A / H7N7|EPI1902725
A/bar-tailed godwit/Liaoning/DD337/2019 H10N7 |2019-04-26|PA|A / H10N7|EPI2436381
A/Wild bird feces/korea/H337/2018|2018-12-12|PA|A / H7N5|EPI2113362
A/duck/Taipei/18WB0137-6-10-E1/2018|2018-12-18|PA|A / H7N1|EPI3406888
A/mallard/Anhui/3-617/2019|2019-11-19|PA|A / H6N1|EPI1743664
A/duck/Kaohsiung/18WB0131-5/2018|2018-12-01|PA|A / H7N7|EPI3407360
A/Mallard/Russia Primorje/153/2019|2019-10-06|PA|A / H4N6|EPI1656997
A/duck/Bangladesh/38305/2019|2019-02-09|PA|A / H11N3|EPI1778187
A/Wild bird/South Korea/Q19-1707/2019|2019-12-03|PA|A / H10N1|EPI3039199
A/wild bird/Toyama/161201/2021|2021-12-20|PA|A / H11N9|EPI2885603
A/Common Teal Anas crecca /South Korea/KNU2021-22/2021|2021-02-17|PA|A / H8N4|EPI2153539
A/environment/Japan/KU-H16/2021|2021-01-25|PA|A / H1N1|EPI2596194
A/duck/Bangladesh/45997/2020|2020-10-20|PA|A / H1N3|EPI2147552
A/duck/Bangladesh/44493/2020|2020-02-22|PA|A / H9N9|EPI1905030
A/common teal/Novosibirsk region/3515k/2020|2020-08-29|PA|A / H3N8|EPI1850416
A/duck/Bangladesh/18D1577/2021|2021-01-04|PA|A / H3N2|EPI1985987
A/environment/Bangladesh/52224/2022|2022-01-30|PA|A / H10N4|EPI2201637
A/goose/China/KUST-ZT-BTY5/2021|2021-12-01|PA|A / H5N1|EPI2882890
A/duck/Tottori/311215/2020|2020-12-15|PA|A / H5N2|EPI1930522
A/Wildbird/Bangladesh/WB-015-T/2023|2023-01-26|PA|A / H5N1|EPI3116466
A/Mandarin duck/Korea/24WS005-2 H5N1 |2024-10-14| PA
A/Wild bird/South Korea/K23-1371-3/2023|2023-12-21|PA|A / H4N6|EPI3040195
A/duck/Bangladesh/17-D-2338/2024|2024-02-22|PA|A / H5N1|EPI3355192
A/Zhejiang/CNIC-ZJU01/2023|2023-12-15|PA|A / H10N5|EPI2970795
A/duck/Bangladesh/19-D-2266/2023|2023-11-22|PA|A / H4N6|EPI3358787
A/wild migratory bird/Bangladesh/WB-142-C/2024|2024-02-11|PA|A / H12N5|EPI3354540
A/duck/Bangladesh/WF-504/2024|2024-01-22|PA|A / H7N4|EPI3354475
A/gadwall/Chany/893/2018|2018-10-19|PA|A / H3N8|EPI1333762
A/duck/Bangladesh/37509/2019|2019-01-17|PA|A / H8N4|EPI1778068
A/common teal/Chany Lake/213/2020|2020-08-29|PA|A / H5N2|EPI1846412
A/duck/Mongolia/447/2018|2018-09-02|PA|A / H4N6|EPI1818099
A/egret/Anhui/A1-267/2020|2020-10-01|PA|A / H3N8|EPI2550817
A/swan/Shandong/W4322/2020|2020-11-23|PA|A / H10N4|EPI2146817
A/garganey/Bangladesh/38920/2019|2019-02-07|PA|A / H7N4|EPI1778058
A/environment/Kagoshima/KU-C6/2021 H4N2 |2021-12-13|PA|A / H4N2|EPI2553124
A/duck/Tottori/NK12F18/2021|2021-12-01|PA|A / H7N7|EPI3327086
A/duck/Tochigi/090114/2022|2022-01-18|PA|A / H10N7|EPI2885801
A/mallard/Yakutia/47/2020|2020-08-13|PA|A / H7N7|EPI1848199
A/mallard/Korea/KNU-24/2023|2023-03-15|PA|A / H11N9|EPI2873297
A/Em/Korea/22WF191-23P/2022|2022-11-16|PA|A / H5N1|EPI2742575
A/environment/Kagoshima/KU-C8/2023 H3N6 |2023-12-19|PA|A / H3N6|EPI2917650
A/Wild bird/South Korea/Q23-324-4/2023|2023-10-18|PA|A / H3N8|EPI3040267
A/chicken/Miyazaki/22C1T/2023|2023-01-10|PA|A / H5N1|EPI2751864
A/egret/Korea/22WC188/2022|2022-12-09|PA|A / H5N1|EPI2742767
A/Em/Korea/22WF123-24P/2022|2022-11-02|PA|A / H5N1|EPI2742423
A/spot billed/Korea/KNU-11/2022|2022-02-09|PA|A / H7N7|EPI2873188
A/Swan/Shandong/W4154/2021|2021-12-23|PA|A / H11N3|EPI3217118
A/environment/Kagoshima/KU-E2/2023 H3N2 |2023-11-13|PA|A / H3N2|EPI2917584
A/Greater white-fronted goose/South Korea/K23-1027-4/2023|2023-11-10|PA|A / H9N2|EPI3040155
A/duck/Bangladesh/18D1813/2022|2022-01-10|PA|A / H5N3|EPI3115797
A/whooper swan/Shanxi/SX126/2020|2020-11-10|PA|A / H9N2|EPI1858881
A/duck/Bangladesh/17D1968/2022|2022-08-18|PA|A / H3N6|EPI3115922
A/Duck/Bangladesh/55253/2022|2022-08-07|PA|A / H4N6|EPI2376685

HA

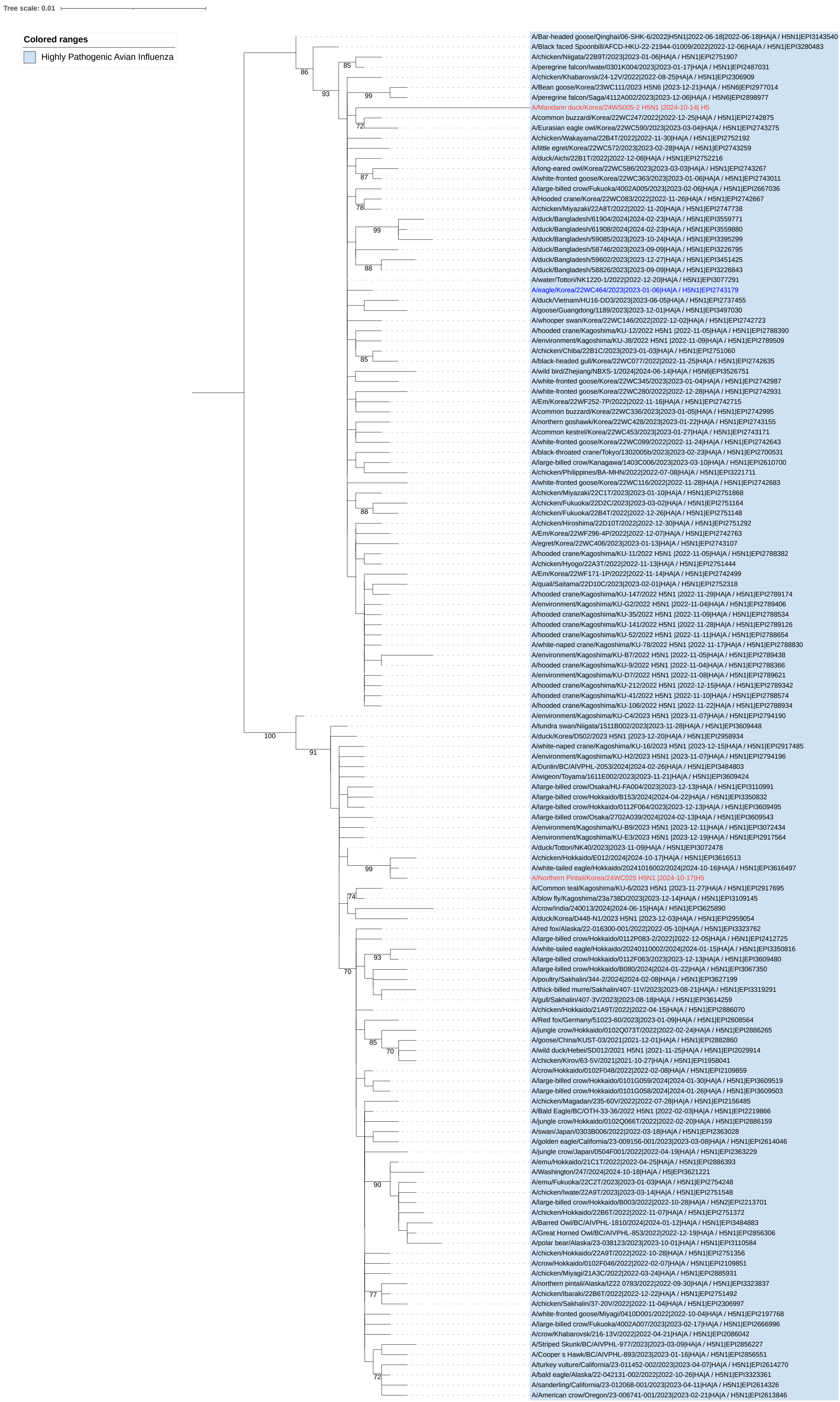
Tree scale: 0.01
Colored ranges
Highly Pathogenic Avian Influenza
A/Bar-headed goose/Qinghai/06-SHK-6/2022|H5N1|2022-06-18|2022-06-18|HA|A / H5N1|EPI3143540
A/Black faced Spoonbill/AFCD-HKU-22-21944-01009/2022|2022-12-06|HA|A / H5N1|EPI3280483
A/chicken/Niigata/22B9T/2023|2023-01-06|HA|A / H5N1|EPI2751907
A/peregrine falcon/Iwate/0301K004/2023|2023-01-17|HA|A / H5N1|EPI2487031
A/chicken/Khabarovsk/24-12V/2022|2022-08-25|HA|A / H5N1|EPI2306909
A/Bean goose/Korea/23WC111/2023 H5N6 |2023-12-21|HA|A / H5N6|EPI2977014
A/peregrine falcon/Saga/4112A002/2023|2023-12-06|HA|A / H5N6|EPI2898977
A/Mandarin duck/Korea/24WS005-2 H5N1 |2024-10-14| H5
A/common buzzard/Korea/22WC247/2022|2022-12-25|HA|A / H5N1|EPI2742875
A/Eurasian eagle owl/Korea/22WC590/2023|2023-03-04|HA|A / H5N1|EPI2743275
A/chicken/Wakayama/22B4T/2022|2022-11-30|HA|A / H5N1|EPI2752192
A/little egret/Korea/22WC572/2023|2023-02-28|HA|A / H5N1|EPI2743259
A/duck/Aichi/22B1T/2022|2022-12-08|HA|A / H5N1|EPI2752216
A/long-eared owl/Korea/22WC586/2023|2023-03-03|HA|A / H5N1|EPI2743267
A/white-fronted goose/Korea/22WC363/2023|2023-01-06|HA|A / H5N1|EPI2743011
A/large-billed crow/Fukuoka/4002A005/2023|2023-02-06|HA|A / H5N1|EPI2667036
A/Hooded crane/Korea/22WC083/2022|2022-11-26|HA|A / H5N1|EPI2742667
A/chicken/Miyazaki/22A8T/2022|2022-11-20|HA|A / H5N1|EPI2747738
A/duck/Bangladesh/61904/2024|2024-02-23|HA|A / H5N1|EPI3559771
A/duck/Bangladesh/61908/2024|2024-02-23|HA|A / H5N1|EPI3559880
A/duck/Bangladesh/59085/2023|2023-10-24|HA|A / H5N1|EPI3395299
A/duck/Bangladesh/58746/2023|2023-09-09|HA|A / H5N1|EPI3226795
A/duck/Bangladesh/59602/2023|2023-12-27|HA|A / H5N1|EPI3451425
A/duck/Bangladesh/58826/2023|2023-09-09|HA|A / H5N1|EPI3226843
A/water/Tottori/NK1220-1/2022|2022-12-20|HA|A / H5N1|EPI3077291
A/eagle/Korea/22WC464/2023|2023-01-06|HA|A / H5N1|EPI2743179
A/duck/Vietnam/HU16-DD3/2023|2023-06-05|HA|A / H5N1|EPI2737455
A/goose/Guangdong/1189/2023|2023-12-01|HA|A / H5N1|EPI3497030
A/whooper swan/Korea/22WC146/2022|2022-12-02|HA|A / H5N1|EPI2742723
A/hooded crane/Kagoshima/KU-12/2022 H5N1 |2022-11-05|HA|A / H5N1|EPI2788390
A/environment/Kagoshima/KU-J8/2022 H5N1 |2022-11-09|HA|A / H5N1|EPI2789509
A/chicken/Chiba/22B1C/2023|2023-01-03|HA|A / H5N1|EPI2751060
A/black-headed gull/Korea/22WC077/2022|2022-11-25|HA|A / H5N1|EPI2742635
A/wild bird/Zhejiang/NBXS-1/2024|2024-06-14|HA|A / H5N6|EPI3526751
A/white-fronted goose/Korea/22WC345/2023|2023-01-04|HA|A / H5N1|EPI2742987
A/white-fronted goose/Korea/22WC280/2022|2022-12-28|HA|A / H5N1|EPI2742931
A/Em/Korea/22WF252-7P/2022|2022-11-16|HA|A / H5N1|EPI2742715
A/common buzzard/Korea/22WC336/2023|2023-01-05|HA|A / H5N1|EPI2742995
A/northern goshawk/Korea/22WC428/2023|2023-01-22|HA|A / H5N1|EPI2743155
A/common kestrel/Korea/22WC453/2023|2023-01-27|HA|A / H5N1|EPI2743171
A/white-fronted goose/Korea/22WC099/2022|2022-11-24|HA|A / H5N1|EPI2742643
A/black-throated crane/Tokyo/1302005b/2023|2023-02-23|HA|A / H5N1|EPI2700531
A/large-billed crow/Kanagawa/1403C006/2023|2023-03-10|HA|A / H5N1|EPI2610700
A/chicken/Philippines/BA-MHN/2022|2022-07-08|HA|A / H5N1|EPI3221711
A/white-fronted goose/Korea/22WC116/2022|2022-11-28|HA|A / H5N1|EPI2742683
A/chicken/Miyazaki/22C1T/2023|2023-01-10|HA|A / H5N1|EPI2751868
A/chicken/Fukuoka/22D2C/2023|2023-03-02|HA|A / H5N1|EPI2751164
A/chicken/Fukuoka/22B4T/2022|2022-12-26|HA|A / H5N1|EPI2751148
A/chicken/Hiroshima/22D10T/2022|2022-12-30|HA|A / H5N1|EPI2751292
A/Em/Korea/22WF296-4P/2022|2022-12-07|HA|A / H5N1|EPI2742763
A/egret/Korea/22WC406/2023|2023-01-13|HA|A / H5N1|EPI2743107
A/hooded crane/Kagoshima/KU-11/2022 H5N1 |2022-11-05|HA|A / H5N1|EPI2788382
A/chicken/Hyogo/22A3T/2022|2022-11-13|HA|A / H5N1|EPI2751444
A/Em/Korea/22WF171-1P/2022|2022-11-14|HA|A / H5N1|EPI2742499
A/quail/Saitama/22D10C/2023|2023-02-01|HA|A / H5N1|EPI2752318
A/hooded crane/Kagoshima/KU-147/2022 H5N1 |2022-11-29|HA|A / H5N1|EPI2789174
A/environment/Kagoshima/KU-G2/2022 H5N1 |2022-11-04|HA|A / H5N1|EPI2789406
A/hooded crane/Kagoshima/KU-35/2022 H5N1 |2022-11-09|HA|A / H5N1|EPI2788534
A/hooded crane/Kagoshima/KU-141/2022 H5N1 |2022-11-28|HA|A / H5N1|EPI2789126
A/hooded crane/Kagoshima/KU-52/2022 H5N1 |2022-11-11|HA|A / H5N1|EPI2788654
A/white-naped crane/Kagoshima/KU-78/2022 H5N1 |2022-11-17|HA|A / H5N1|EPI2788830
A/environment/Kagoshima/KU-B7/2022 H5N1 |2022-11-05|HA|A / H5N1|EPI2789438
A/hooded crane/Kagoshima/KU-9/2022 H5N1 |2022-11-04|HA|A / H5N1|EPI2788366
A/environment/Kagoshima/KU-D7/2022 H5N1 |2022-11-08|HA|A / H5N1|EPI2789621
A/hooded crane/Kagoshima/KU-212/2022 H5N1 |2022-12-15|HA|A / H5N1|EPI2789342
A/hooded crane/Kagoshima/KU-41/2022 H5N1 |2022-11-10|HA|A / H5N1|EPI2788574
A/hooded crane/Kagoshima/KU-106/2022 H5N1 |2022-11-22|HA|A / H5N1|EPI2788934
A/environment/Kagoshima/KU-C4/2023 H5N1 |2023-11-07|HA|A / H5N1|EPI2794190
A/tundra swan/Niigata/1511B002/2023|2023-11-28|HA|A / H5N1|EPI3609448
A/duck/Korea/D502/2023 H5N1 |2023-12-20|HA|A / H5N1|EPI2958934
A/white-naped crane/Kagoshima/KU-16/2023 H5N1 |2023-12-15|HA|A / H5N1|EPI2917485
A/environment/Kagoshima/KU-H2/2023 H5N1 |2023-11-07|HA|A / H5N1|EPI2794196
A/Dunlin/BC/AIVPHL-2053/2024|2024-02-26|HA|A / H5N1|EPI3484803
A/wigeon/Toyama/1611E002/2023|2023-11-21|HA|A / H5N1|EPI3609424
A/large-billed crow/Osaka/HU-FA004/2023|2023-12-13|HA|A / H5N1|EPI3110991
A/large-billed crow/Hokkaido/B153/2024|2024-04-22|HA|A / H5N1|EPI3350832
A/large-billed crow/Hokkaido/0112F064/2023|2023-12-13|HA|A / H5N1|EPI3609495
A/large-billed crow/Osaka/2702A039/2024|2024-02-13|HA|A / H5N1|EPI3609543
A/environment/Kagoshima/KU-B9/2023 H5N1 |2023-12-11|HA|A / H5N1|EPI3072434
A/environment/Kagoshima/KU-E3/2023 H5N1 |2023-12-19|HA|A / H5N1|EPI2917564
A/duck/Tottori/NK40/2023|2023-11-09|HA|A / H5N1|EPI3072478
A/chicken/Hokkaido/E012/2024|2024-10-17|HA|A / H5N1|EPI3616513
A/white-tailed eagle/Hokkaido/20241016002/2024|2024-10-16|HA|A / H5N1|EPI3616497
A/Northern Pintail/Korea/24WC025 H5N1 |2024-10-17|H5
A/Common teal/Kagoshima/KU-6/2023 H5N1 |2023-11-27|HA|A / H5N1|EPI2917695
A/blow fly/Kagoshima/23a738D/2023|2023-12-14|HA|A / H5N1|EPI3109145
A/crow/India/240013/2024|2024-06-15|HA|A / H5N1|EPI3625890
A/duck/Korea/D448-N1/2023 H5N1 |2023-12-03|HA|A / H5N1|EPI2959054
A/red fox/Alaska/22-016300-001/2022|2022-05-10|HA|A / H5N1|EPI3323762
A/large-billed crow/Hokkaido/0112P083-2/2022|2022-12-05|HA|A / H5N1|EPI2412725
A/white-tailed eagle/Hokkaido/20240110002/2024|2024-01-15|HA|A / H5N1|EPI3350816
A/large-billed crow/Hokkaido/0112F063/2023|2023-12-13|HA|A / H5N1|EPI3609480
A/large-billed crow/Hokkaido/B080/2024|2024-01-22|HA|A / H5N1|EPI3067350
A/poultry/Sakhalin/344-2/2024|2024-02-08|HA|A / H5N1|EPI3627199
A/thick-billed murre/Sakhalin/407-11V/2023|2023-08-21|HA|A / H5N1|EPI3319291
A/gull/Sakhalin/407-3V/2023|2023-08-18|HA|A / H5N1|EPI3614259
A/chicken/Hokkaido/21A9T/2022|2022-04-15|HA|A / H5N1|EPI2886070
A/Red fox/Germany/51023-60/2023|2023-01-09|HA|A / H5N1|EPI2608564
A/jungle crow/Hokkaido/0102Q073T/2022|2022-02-24|HA|A / H5N1|EPI2886265
A/goose/China/KUST-03/2021|2021-12-01|HA|A / H5N1|EPI2882860
A/wild duck/Hebei/SD012/2021 H5N1 |2021-11-25|HA|A / H5N1|EPI2029914
A/chicken/Kirov/63-5V/2021|2021-10-27|HA|A / H5N1|EPI1958041
A/crow/Hokkaido/0102F048/2022|2022-02-08|HA|A / H5N1|EPI2109859
A/large-billed crow/Hokkaido/0101G059/2024|2024-01-30|HA|A / H5N1|EPI3609519
A/large-billed crow/Hokkaido/0101G058/2024|2024-01-26|HA|A / H5N1|EPI3609503
A/chicken/Magadan/235-60V/2022|2022-07-28|HA|A / H5N1|EPI2156485
A/Bald Eagle/BC/OTH-33-36/2022 H5N1 |2022-02-03|HA|A / H5N1|EPI2219866
A/jungle crow/Hokkaido/0102Q066T/2022|2022-02-20|HA|A / H5N1|EPI2886159
A/swan/Japan/0303B006/2022|2022-03-18|HA|A / H5N1|EPI2363028
A/golden eagle/California/23-009156-001/2023|2023-03-08|HA|A / H5N1|EPI2614046
A/jungle crow/Japan/0504F001/2022|2022-04-19|HA|A / H5N1|EPI2363229
A/emu/Hokkaido/21C1T/2022|2022-04-25|HA|A / H5N1|EPI2886393
A/Washington/247/2024|2024-10-18|HA|A / H5|EPI3621221
A/emu/Fukuoka/22C2T/2023|2023-01-03|HA|A / H5N1|EPI2754248
A/chicken/Iwate/22A9T/2023|2023-03-14|HA|A / H5N1|EPI2751548
A/large-billed crow/Hokkaido/B003/2022|2022-10-28|HA|A / H5N2|EPI2213701
A/chicken/Hokkaido/22B6T/2022|2022-11-07|HA|A / H5N1|EPI2751372
A/Barred Owl/BC/AIVPHL-1810/2024|2024-01-12|HA|A / H5N1|EPI3484883
A/Great Horned Owl/BC/AIVPHL-853/2022|2022-12-19|HA|A / H5N1|EPI2856306
A/polar bear/Alaska/23-038123/2023|2023-10-01|HA|A / H5N1|EPI3110584
A/chicken/Hokkaido/22A9T/2022|2022-10-28|HA|A / H5N1|EPI2751356
A/crow/Hokkaido/0102F046/2022|2022-02-07|HA|A / H5N1|EPI2109851
A/chicken/Miyagi/21A3C/2022|2022-03-24|HA|A / H5N1|EPI2885931
A/northern pintail/Alaska/IZ22 0783/2022|2022-09-30|HA|A / H5N1|EPI3323837
A/chicken/Ibaraki/22B6T/2022|2022-12-22|HA|A / H5N1|EPI2751492
A/chicken/Sakhalin/37-20V/2022|2022-11-04|HA|A / H5N1|EPI2306997
A/white-fronted goose/Miyagi/0410D001/2022|2022-10-04|HA|A / H5N1|EPI2197768
A/large-billed crow/Fukuoka/4002A007/2023|2023-02-17|HA|A / H5N1|EPI2666996
A/crow/Khabarovsk/216-13V/2022|2022-04-21|HA|A / H5N1|EPI2086042
A/Striped Skunk/BC/AIVPHL-977/2023|2023-03-09|HA|A / H5N1|EPI2856227
A/Cooper s Hawk/BC/AIVPHL-893/2023|2023-01-16|HA|A / H5N1|EPI2856551
A/turkey vulture/California/23-011452-002/2023|2023-04-07|HA|A / H5N1|EPI2614270
A/bald eagle/Alaska/22-042131-002/2022|2022-10-26|HA|A / H5N1|EPI3323361
A/sanderling/California/23-012068-001/2023|2023-04-11|HA|A / H5N1|EPI2614326
A/American crow/Oregon/23-006741-001/2023|2023-02-21|HA|A / H5N1|EPI2613846

NP

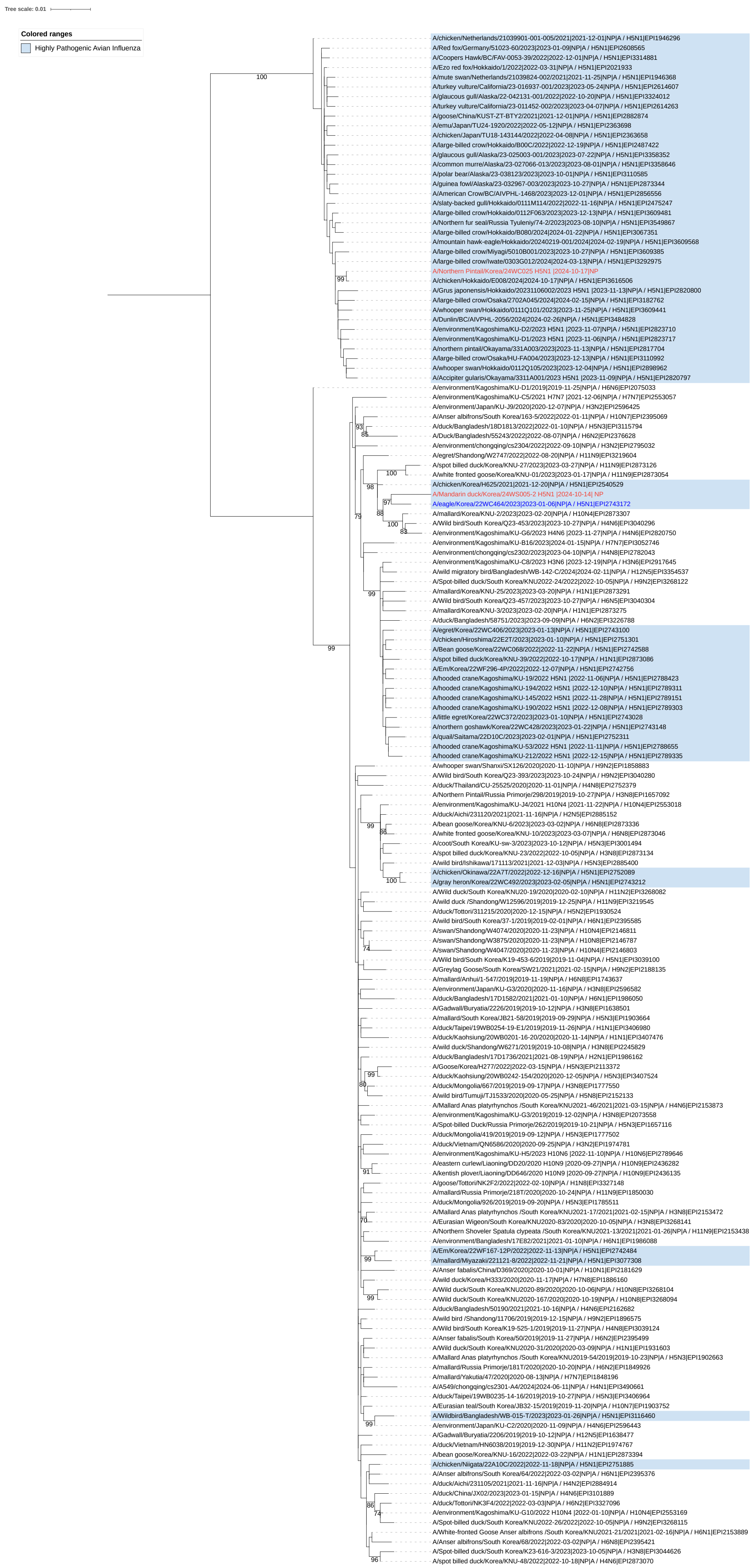
Tree scale: 0.01
Colored ranges
Highly Pathogenic Avian Influenza
A/chicken/Netherlands/21039901-001-005/2021|2021-12-01|NP|A / H5N1|EPI1946296
A/Red fox/Germany/51023-60/2023|2023-01-09|NP|A / H5N1|EPI2608565
A/Coopers Hawk/BC/FAV-0053-39/2022|2022-12-01|NP|A / H5N1|EPI3314881
A/Ezo red fox/Hokkaido/1/2022|2022-03-31|NP|A / H5N1|EPI2021933
A/mute swan/Netherlands/21039824-002/2021|2021-11-25|NP|A / H5N1|EPI1946368
A/turkey vulture/California/23-016937-001/2023|2023-05-24|NP|A / H5N1|EPI2614607
A/glaucous gull/Alaska/22-042131-001/2022|2022-12-01|NP|A / H5N1|EPI3324012
A/turkey vulture/California/23-011452-002/2023|2023-04-07|NP|A / H5N1|EPI2614263
A/goose/China/KUST-ZT-BTY2/2021|2021-12-01|NP|A / H5N1|EPI2882874
A/emu/Japan/TU24-1920/2022|2022-05-12|NP|A / H5N1|EPI2363698
A/chicken/Japan/TU18-143144/2022|2022-04-08|NP|A / H5N1|EPI2363658
A/large-billed crow/Hokkaido/B00C/2022|2022-12-19|NP|A / H5N1|EPI2487422
A/glaucous gull/Alaska/23-025003-001/2023|2023-07-22|NP|A / H5N1|EPI3358352
A/common murre/Alaska/23-027066-013/2023|2023-08-01|NP|A / H5N1|EPI3358646
A/polar bear/Alaska/23-038123/2023|2023-10-01|NP|A / H5N1|EPI3110585
A/guinea fowl/Alaska/23-032967-003/2023|2023-10-27|NP|A / H5N1|EPI2873344
A/American Crow/BC/AIVPHL-1468/2023|2023-12-01|NP|A / H5N1|EPI2856556
A/slaty-backed gull/Hokkaido/0111M114/2022|2022-11-16|NP|A / H5N1|EPI2475247
A/large-billed crow/Hokkaido/0112F063/2023|2023-12-13|NP|A / H5N1|EPI3609481
A/Northern fur seal/Russia Tyuleniy/74-2/2023|2023-08-10|NP|A / H5N1|EPI3549867
A/large-billed crow/Hokkaido/B080/2024|2024-01-22|NP|A / H5N1|EPI3067351
A/mountain hawk-eagle/Hokkaido/20240219-001/2024|2024-02-19|NP|A / H5N1|EPI3609568
A/large-billed crow/Miyagi/5010B001/2023|2023-10-27|NP|A / H5N1|EPI3609385
A/large-billed crow/Iwate/0303G012/2024|2024-03-13|NP|A / H5N1|EPI3292975
A/Northern Pintail/Korea/24WC025 H5N1 |2024-10-17| NP
A/chicken/Hokkaido/E008/2024|2024-10-17|NP|A / H5N1|EPI3616506
A/Grus japonensis/Hokkaido/20231106002/2023 H5N1 |2023-11-13|NP|A / H5N1|EPI2820800
A/large-billed crow/Osaka/2702A045/2024|2024-02-15|NP|A / H5N1|EPI3182762
A/whooper swan/Hokkaido/0111Q101/2023|2023-11-25|NP|A / H5N1|EPI3609441
A/Dunlin/BC/AIVPHL-2056/2024|2024-02-26|NP|A / H5N1|EPI3484828
A/environment/Kagoshima/KU-D2/2023 H5N1 |2023-11-07|NP|A / H5N1|EPI2823710
A/environment/Kagoshima/KU-D1/2023 H5N1 |2023-11-06|NP|A / H5N1|EPI2823717
A/northern pintail/Okayama/331A003/2023|2023-11-13|NP|A / H5N1|EPI2817704
A/large-billed crow/Osaka/HU-FA004/2023|2023-12-13|NP|A / H5N1|EPI3110992
A/whooper swan/Hokkaido/0112Q105/2023|2023-12-04|NP|A / H5N1|EPI2898962
A/Accipiter gularis/Okayama/3311A001/2023 H5N1 |2023-11-09|NP|A / H5N1|EPI2820797
A/environment/Kagoshima/KU-D1/2019|2019-11-25|NP|A / H6N6|EPI2075033
A/environment/Kagoshima/KU-C5/2021 H7N7 |2021-12-06|NP|A / H7N7|EPI2553057
A/environment/Japan/KU-J9/2020|2020-12-07|NP|A / H3N2|EPI2596425
A/Anser albifrons/South Korea/163-5/2022|2022-01-11|NP|A / H10N7|EPI2395069
A/duck/Bangladesh/18D1813/2022|2022-01-10|NP|A / H5N3|EPI3115794
A/Duck/Bangladesh/55243/2022|2022-08-07|NP|A / H6N2|EPI2376628
A/environment/chongqing/cs2304/2022|2022-09-10|NP|A / H3N2|EPI2795032
A/egret/Shandong/W2747/2022|2022-08-20|NP|A / H11N9|EPI3219604
A/spot billed duck/Korea/KNU-27/2023|2023-03-27|NP|A / H11N9|EPI2873126
A/white fronted goose/Korea/KNU-01/2023|2023-01-17|NP|A / H11N9|EPI2873054
A/chicken/Korea/H625/2021|2021-12-20|NP|A / H5N1|EPI2540529
A/Mandarin duck/Korea/24WS005-2 H5N1 |2024-10-14| NP
A/eagle/Korea/22WC464/2023|2023-01-06|NP|A / H5N1|EPI2743172
A/mallard/Korea/KNU-2/2023|2023-02-20|NP|A / H10N4|EPI2873307
A/Wild bird/South Korea/Q23-453/2023|2023-10-27|NP|A / H4N6|EPI3040296
A/environment/Kagoshima/KU-G6/2023 H4N6 |2023-11-27|NP|A / H4N6|EPI2820750
A/environment/Kagoshima/KU-B16/2023|2024-01-15|NP|A / H7N7|EPI3052746
A/environment/chongqing/cs2302/2023|2023-04-10|NP|A / H4N8|EPI2782043
A/environment/Kagoshima/KU-C8/2023 H3N6 |2023-12-19|NP|A / H3N6|EPI2917645
A/wild migratory bird/Bangladesh/WB-142-C/2024|2024-02-11|NP|A / H12N5|EPI3354537
A/Spot-billed duck/South Korea/KNU2022-24/2022|2022-10-05|NP|A / H9N2|EPI3268122
A/mallard/Korea/KNU-25/2023|2023-03-20|NP|A / H1N1|EPI2873291
A/Wild bird/South Korea/Q23-457/2023|2023-10-27|NP|A / H6N5|EPI3040304
A/mallard/Korea/KNU-3/2023|2023-02-20|NP|A / H1N1|EPI2873275
A/duck/Bangladesh/58751/2023|2023-09-09|NP|A / H6N2|EPI3226788
A/egret/Korea/22WC406/2023|2023-01-13|NP|A / H5N1|EPI2743100
A/chicken/Hiroshima/22E2T/2023|2023-01-10|NP|A / H5N1|EPI2751301
A/Bean goose/Korea/22WC068/2022|2022-11-22|NP|A / H5N1|EPI2742588
A/spot billed duck/Korea/KNU-39/2022|2022-10-17|NP|A / H1N1|EPI2873086
A/Em/Korea/22WF296-4P/2022|2022-12-07|NP|A / H5N1|EPI2742756
A/hooded crane/Kagoshima/KU-19/2022 H5N1 |2022-11-06|NP|A / H5N1|EPI2788423
A/hooded crane/Kagoshima/KU-194/2022 H5N1 |2022-12-10|NP|A / H5N1|EPI2789311
A/hooded crane/Kagoshima/KU-145/2022 H5N1 |2022-11-28|NP|A / H5N1|EPI2789151
A/hooded crane/Kagoshima/KU-190/2022 H5N1 |2022-12-08|NP|A / H5N1|EPI2789303
A/little egret/Korea/22WC372/2023|2023-01-10|NP|A / H5N1|EPI2743028
A/northern goshawk/Korea/22WC428/2023|2023-01-22|NP|A / H5N1|EPI2743148
A/quail/Saitama/22D10C/2023|2023-02-01|NP|A / H5N1|EPI2752311
A/hooded crane/Kagoshima/KU-53/2022 H5N1 |2022-11-11|NP|A / H5N1|EPI2788655
A/hooded crane/Kagoshima/KU-212/2022 H5N1 |2022-12-15|NP|A / H5N1|EPI2789335
A/whooper swan/Shanxi/SX126/2020|2020-11-10|NP|A / H9N2|EPI1858883
A/Wild bird/South Korea/Q23-393/2023|2023-10-24|NP|A / H9N2|EPI3040280
A/duck/Thailand/CU-25525/2020|2020-11-01|NP|A / H4N8|EPI2752379
A/Northern Pintail/Russia Primorje/298/2019|2019-10-27|NP|A / H3N8|EPI1657092
A/environment/Kagoshima/KU-J4/2021 H10N4 |2021-11-22|NP|A / H10N4|EPI2553018
A/duck/Aichi/231120/2021|2021-11-16|NP|A / H2N5|EPI2885152
A/bean goose/Korea/KNU-6/2023|2023-03-02|NP|A / H6N8|EPI2873336
A/white fronted goose/Korea/KNU-10/2023|2023-03-07|NP|A / H6N8|EPI2873046
A/coot/South Korea/KU-sw-3/2023|2023-10-12|NP|A / H5N3|EPI3001494
A/spot billed duck/Korea/KNU-23/2022|2022-10-05|NP|A / H3N8|EPI2873134
A/wild bird/Ishikawa/171113/2021|2021-12-03|NP|A / H5N3|EPI2885400
A/chicken/Okinawa/22A7T/2022|2022-12-16|NP|A / H5N1|EPI2752089
A/gray heron/Korea/22WC492/2023|2023-02-05|NP|A / H5N1|EPI2743212
A/Wild duck/South Korea/KNU20-19/2020|2020-02-10|NP|A / H11N2|EPI3268082
A/wild duck /Shandong/W12596/2019|2019-12-25|NP|A / H11N9|EPI3219545
A/duck/Tottori/311215/2020|2020-12-15|NP|A / H5N2|EPI1930524
A/wild bird/South Korea/37-1/2019|2019-02-01|NP|A / H6N1|EPI2395585
A/swan/Shandong/W4074/2020|2020-11-23|NP|A / H10N4|EPI2146811
A/swan/Shandong/W3875/2020|2020-11-23|NP|A / H10N8|EPI2146787
A/swan/Shandong/W4047/2020|2020-11-23|NP|A / H10N4|EPI2146803
A/Wild bird/South Korea/K19-453-6/2019|2019-11-04|NP|A / H5N1|EPI3039100
A/Greylag Goose/South Korea/SW21/2021|2021-02-15|NP|A / H9N2|EPI2188135
A/mallard/Anhui/1-547/2019|2019-11-19|NP|A / H6N8|EPI1743637
A/environment/Japan/KU-G3/2020|2020-11-16|NP|A / H3N8|EPI2596582
A/duck/Bangladesh/17D1582/2021|2021-01-10|NP|A / H6N1|EPI1986050
A/Gadwall/Buryatia/2226/2019|2019-10-12|NP|A / H3N8|EPI1638501
A/mallard/South Korea/JB21-58/2019|2019-09-29|NP|A / H5N3|EPI1903664
A/duck/Taipei/19WB0254-19-E1/2019|2019-11-26|NP|A / H1N1|EPI3406980
A/duck/Kaohsiung/20WB0201-16-20/2020|2020-11-14|NP|A / H1N1|EPI3407476
A/wild duck/Shandong/W6271/2019|2019-10-08|NP|A / H3N8|EPI2245829
A/duck/Bangladesh/17D1736/2021|2021-08-19|NP|A / H2N1|EPI1986162
A/Goose/Korea/H277/2022|2022-03-15|NP|A / H5N3|EPI2113372
A/duck/Kaohsiung/20WB0242-154/2020|2020-12-05|NP|A / H5N3|EPI3407524
A/duck/Mongolia/667/2019|2019-09-17|NP|A / H3N8|EPI1777550
A/wild bird/Tumuji/TJ1533/2020|2020-05-25|NP|A / H5N8|EPI2152133
A/Mallard Anas platyrhynchos /South Korea/KNU2021-46/2021|2021-03-15|NP|A / H4N6|EPI2153873
A/environment/Kagoshima/KU-G3/2019|2019-12-02|NP|A / H3N8|EPI2073558
A/Spot-billed Duck/Russia Primorje/262/2019|2019-10-21|NP|A / H5N3|EPI1657116
A/duck/Mongolia/419/2019|2019-09-12|NP|A / H5N3|EPI1777502
A/duck/Vietnam/QN6586/2020|2020-09-25|NP|A / H3N2|EPI1974781
A/environment/Kagoshima/KU-H5/2023 H10N6 |2022-11-10|NP|A / H10N6|EPI2789646
A/eastern curlew/Liaoning/DD20/2020 H10N9 |2020-09-27|NP|A / H10N9|EPI2436282
A/kentish plover/Liaoning/DD646/2020 H10N9 |2020-09-27|NP|A / H10N9|EPI2436135
A/goose/Tottori/NK2F2/2022|2022-02-10|NP|A / H1N8|EPI3327148
A/mallard/Russia Primorje/218T/2020|2020-10-24|NP|A / H11N9|EPI1850030
A/duck/Mongolia/926/2019|2019-09-20|NP|A / H5N3|EPI1785511
A/Mallard Anas platyrhynchos /South Korea/KNU2021-17/2021|2021-02-15|NP|A / H3N8|EPI2153472
A/Eurasian Wigeon/South Korea/KNU2020-83/2020|2020-10-05|NP|A / H3N8|EPI3268141
A/Northern Shoveler Spatula clypeata /South Korea/KNU2021-13/2021|2021-01-26|NP|A / H11N9|EPI2153438
A/environment/Bangladesh/17E82/2021|2021-01-10|NP|A / H6N1|EPI1986088
A/Em/Korea/22WF167-12P/2022|2022-11-13|NP|A / H5N1|EPI2742484
A/mallard/Miyazaki/221121-8/2022|2022-11-21|NP|A / H5N1|EPI3077308
A/Anser fabalis/China/D369/2020|2020-10-01|NP|A / H10N1|EPI2181629
A/wild duck/Korea/H333/2020|2020-11-17|NP|A / H7N8|EPI1886160
A/Wild duck/South Korea/KNU2020-89/2020|2020-10-06|NP|A / H10N8|EPI3268104
A/Wild duck/South Korea/KNU2020-167/2020|2020-10-19|NP|A / H10N8|EPI3268094
A/duck/Bangladesh/50190/2021|2021-10-16|NP|A / H4N6|EPI2162682
A/wild bird /Shandong/11706/2019|2019-12-15|NP|A / H9N2|EPI1896575
A/Wild bird/South Korea/K19-525-1/2019|2019-11-27|NP|A / H4N8|EPI3039124
A/Anser fabalis/South Korea/50/2019|2019-11-27|NP|A / H6N2|EPI2395499
A/Wild duck/South Korea/KNU2020-31/2020|2020-03-09|NP|A / H1N1|EPI1931603
A/Mallard Anas platyrhynchos /South Korea/KNU2019-54/2019|2019-10-23|NP|A / H5N3|EPI1902663
A/mallard/Russia Primorje/181T/2020|2020-10-20|NP|A / H6N2|EPI1849926
A/mallard/Yakutia/47/2020|2020-08-13|NP|A / H7N7|EPI1848196
A/A549/chongqing/cs2301-A4/2024|2024-06-11|NP|A / H4N1|EPI3490661
A/duck/Taipei/19WB0235-14-16/2019|2019-10-27|NP|A / H5N3|EPI3406964
A/Eurasian teal/South Korea/JB32-15/2019|2019-11-20|NP|A / H10N7|EPI1903752
A/Wildbird/Bangladesh/WB-015-T/2023|2023-01-26|NP|A / H5N1|EPI3116460
A/environment/Japan/KU-C2/2020|2020-11-09|NP|A / H4N6|EPI2596443
A/Gadwall/Buryatia/2206/2019|2019-10-12|NP|A / H12N5|EPI1638477
A/duck/Vietnam/HN6038/2019|2019-12-30|NP|A / H11N2|EPI1974767
A/bean goose/Korea/KNU-16/2022|2022-03-22|NP|A / H1N1|EPI2873394
A/chicken/Niigata/22A10C/2022|2022-11-18|NP|A / H5N1|EPI2751885
A/Anser albifrons/South Korea/64/2022|2022-03-02|NP|A / H6N1|EPI2395376
A/duck/Aichi/231105/2021|2021-11-16|NP|A / H4N2|EPI2884914
A/duck/China/JX02/2023|2023-01-15|NP|A / H4N6|EPI3101889
A/duck/Tottori/NK3F4/2022|2022-03-03|NP|A / H6N2|EPI3327096
A/environment/Kagoshima/KU-G10/2022 H10N4 |2022-01-10|NP|A / H10N4|EPI2553169
A/Spot-billed duck/South Korea/KNU2022-26/2022|2022-10-05|NP|A / H9N2|EPI3268115
A/White-fronted Goose Anser albifrons /South Korea/KNU2021-21/2021|2021-02-16|NP|A / H6N1|EPI2153889
A/Anser albifrons/South Korea/68/2022|2022-03-02|NP|A / H6N8|EPI2395421
A/Spot-billed duck/South Korea/K23-616-3/2023|2023-10-05|NP|A / H3N8|EPI3044626
A/spot billed duck/Korea/KNU-48/2022|2022-10-18|NP|A / H4N6|EPI2873070

NA

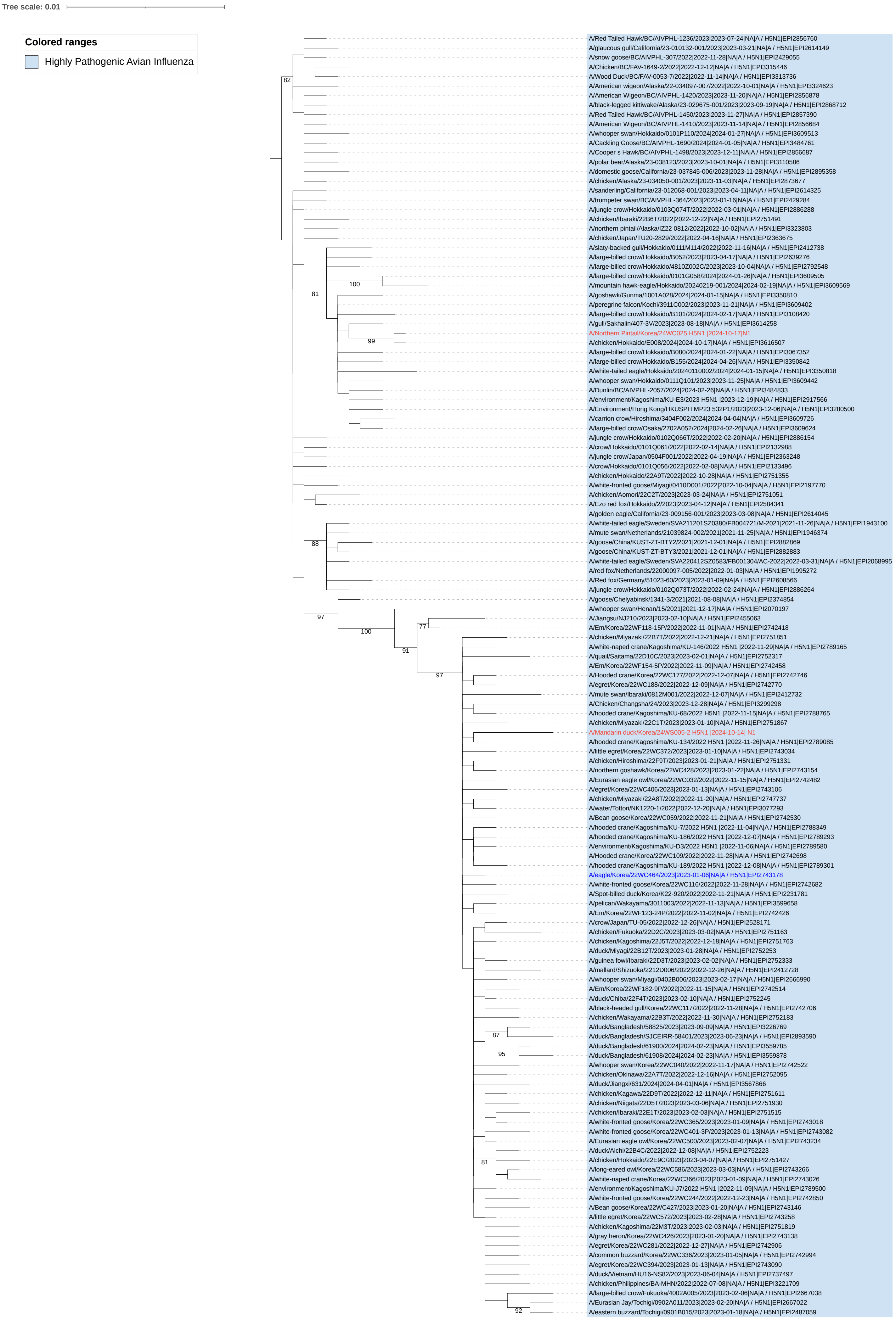

Tree scale: 0.01
Colored ranges
Highly Pathogenic Avian Influenza
82
81
100
99
88
97
100
91
77
97
87
95
81
92
A/Red Tailed Hawk/BC/AIVPHL-1236/2023|2023-07-24|NA|A / H5N1|EPI2856760
A/glaucous gull/California/23-010132-001/2023|2023-03-21|NA|A / H5N1|EPI2614149
A/snow goose/BC/AIVPHL-307/2022|2022-11-28|NA|A / H5N1|EPI2429055
A/Chicken/BC/FAV-1649-2/2022|2022-12-12|NA|A / H5N1|EPI3315446
A/Wood Duck/BC/FAV-0053-7/2022|2022-11-14|NA|A / H5N1|EPI3313736
A/American wigeon/Alaska/22-034097-007/2022|2022-10-01|NA|A / H5N1|EPI3324623
A/American Wigeon/BC/AIVPHL-1420/2023|2023-11-20|NA|A / H5N1|EPI2856878
A/black-legged kittiwake/Alaska/23-029675-001/2023|2023-09-19|NA|A / H5N1|EPI2868712
A/Red Tailed Hawk/BC/AIVPHL-1450/2023|2023-11-27|NA|A / H5N1|EPI2857390
A/American Wigeon/BC/AIVPHL-1410/2023|2023-11-14|NA|A / H5N1|EPI2856684
A/whooper swan/Hokkaido/0101P110/2024|2024-01-27|NA|A / H5N1|EPI3609513
A/Cackling Goose/BC/AIVPHL-1690/2024|2024-01-05|NA|A / H5N1|EPI3484761
A/Cooper s Hawk/BC/AIVPHL-1498/2023|2023-12-11|NA|A / H5N1|EPI2856687
A/polar bear/Alaska/23-038123/2023|2023-10-01|NA|A / H5N1|EPI3110586
A/domestic goose/California/23-037845-006/2023|2023-11-28|NA|A / H5N1|EPI2895358
A/chicken/Alaska/23-034050-001/2023|2023-11-03|NA|A / H5N1|EPI2873677
A/sanderling/California/23-012068-001/2023|2023-04-11|NA|A / H5N1|EPI2614325
A/trumpeter swan/BC/AIVPHL-364/2023|2023-01-16|NA|A / H5N1|EPI2429284
A/jungle crow/Hokkaido/0103Q074T/2022|2022-03-01|NA|A / H5N1|EPI2886288
A/chicken/Ibaraki/22B6T/2022|2022-12-22|NA|A / H5N1|EPI2751491
A/northern pintail/Alaska/IZ22 0812/2022|2022-10-02|NA|A / H5N1|EPI3323803
A/chicken/Japan/TU20-2829/2022|2022-04-16|NA|A / H5N1|EPI2363675
A/slaty-backed gull/Hokkaido/0111M114/2022|2022-11-16|NA|A / H5N1|EPI2412738
A/large-billed crow/Hokkaido/B052/2023|2023-04-17|NA|A / H5N1|EPI2639276
A/large-billed crow/Hokkaido/4810Z002C/2023|2023-10-04|NA|A / H5N1|EPI2792548
A/large-billed crow/Hokkaido/0101G058/2024|2024-01-26|NA|A / H5N1|EPI3609505
A/mountain hawk-eagle/Hokkaido/20240219-001/2024|2024-02-19|NA|A / H5N1|EPI3609569
A/goshawk/Gunma/1001A028/2024|2024-01-15|NA|A / H5N1|EPI3350810
A/peregrine falcon/Kochi/3911C002/2023|2023-11-21|NA|A / H5N1|EPI3609402
A/large-billed crow/Hokkaido/B101/2024|2024-02-17|NA|A / H5N1|EPI3108420
A/gull/Sakhalin/407-3V/2023|2023-08-18|NA|A / H5N1|EPI3614258
A/Northern Pintail/Korea/24WC025 H5N1 |2024-10-17|N1
A/chicken/Hokkaido/E008/2024|2024-10-17|NA|A / H5N1|EPI3616507
A/large-billed crow/Hokkaido/B080/2024|2024-01-22|NA|A / H5N1|EPI3067352
A/large-billed crow/Hokkaido/B155/2024|2024-04-26|NA|A / H5N1|EPI3350842
A/white-tailed eagle/Hokkaido/20240110002/2024|2024-01-15|NA|A / H5N1|EPI3350818
A/whooper swan/Hokkaido/0111Q101/2023|2023-11-25|NA|A / H5N1|EPI3609442
A/Dunlin/BC/AIVPHL-2057/2024|2024-02-26|NA|A / H5N1|EPI3484833
A/environment/Kagoshima/KU-E3/2023 H5N1 |2023-12-19|NA|A / H5N1|EPI2917566
A/Environment/Hong Kong/HKUSPH MP23 532P1/2023|2023-12-06|NA|A / H5N1|EPI3280500
A/carrion crow/Hiroshima/3404F002/2024|2024-04-04|NA|A / H5N1|EPI3609726
A/large-billed crow/Osaka/2702A052/2024|2024-02-26|NA|A / H5N1|EPI3609624
A/jungle crow/Hokkaido/0102Q066T/2022|2022-02-20|NA|A / H5N1|EPI2886154
A/crow/Hokkaido/0101Q061/2022|2022-02-14|NA|A / H5N1|EPI2132988
A/jungle crow/Japan/0504F001/2022|2022-04-19|NA|A / H5N1|EPI2363248
A/crow/Hokkaido/0101Q056/2022|2022-02-08|NA|A / H5N1|EPI2133496
A/chicken/Hokkaido/22A9T/2022|2022-10-28|NA|A / H5N1|EPI2751355
A/white-fronted goose/Miyagi/0410D001/2022|2022-10-04|NA|A / H5N1|EPI2197770
A/chicken/Aomori/22C2T/2023|2023-03-24|NA|A / H5N1|EPI2751051
A/Ezo red fox/Hokkaido/2/2023|2023-04-12|NA|A / H5N1|EPI2584341
A/golden eagle/California/23-009156-001/2023|2023-03-08|NA|A / H5N1|EPI2614045
A/white-tailed eagle/Sweden/SVA211201SZ0380/FB004721/M-2021|2021-11-26|NA|A / H5N1|EPI1943100
A/mute swan/Netherlands/21039824-002/2021|2021-11-25|NA|A / H5N1|EPI1946374
A/goose/China/KUST-ZT-BTY2/2021|2021-12-01|NA|A / H5N1|EPI2882869
A/goose/China/KUST-ZT-BTY3/2021|2021-12-01|NA|A / H5N1|EPI2882883
A/white-tailed eagle/Sweden/SVA220412SZ0583/FB001304/AC-2022|2022-03-31|NA|A / H5N1|EPI2068995
A/red fox/Netherlands/22000097-005/2022|2022-01-03|NA|A / H5N1|EPI1995272
A/Red fox/Germany/51023-60/2023|2023-01-09|NA|A / H5N1|EPI2608566
A/jungle crow/Hokkaido/0102Q073T/2022|2022-02-24|NA|A / H5N1|EPI2886264
A/goose/Chelyabinsk/1341-3/2021|2021-08-08|NA|A / H5N1|EPI2374854
A/whooper swan/Henan/15/2021|2021-12-17|NA|A / H5N1|EPI2070197
A/Jiangsu/NJ210/2023|2023-02-10|NA|A / H5N1|EPI2455063
A/Em/Korea/22WF118-15P/2022|2022-11-01|NA|A / H5N1|EPI2742418
A/chicken/Miyazaki/22B7T/2022|2022-12-21|NA|A / H5N1|EPI2751851
A/white-naped crane/Kagoshima/KU-146/2022 H5N1 |2022-11-29|NA|A / H5N1|EPI2789165
A/quail/Saitama/22D10C/2023|2023-02-01|NA|A / H5N1|EPI2752317
A/Em/Korea/22WF154-5P/2022|2022-11-09|NA|A / H5N1|EPI2742458
A/Hooded crane/Korea/22WC177/2022|2022-12-07|NA|A / H5N1|EPI2742746
A/egret/Korea/22WC188/2022|2022-12-09|NA|A / H5N1|EPI2742770
A/mute swan/Ibaraki/0812M001/2022|2022-12-07|NA|A / H5N1|EPI2412732
A/Chicken/Changsha/24/2023|2023-12-28|NA|A / H5N1|EPI3299298
A/hooded crane/Kagoshima/KU-68/2022 H5N1 |2022-11-15|NA|A / H5N1|EPI2788765
A/chicken/Miyazaki/22C1T/2023|2023-01-10|NA|A / H5N1|EPI2751867
A/Mandarin duck/Korea/24WS005-2 H5N1 |2024-10-14| N1
A/hooded crane/Kagoshima/KU-134/2022 H5N1 |2022-11-26|NA|A / H5N1|EPI2789085
A/little egret/Korea/22WC372/2023|2023-01-10|NA|A / H5N1|EPI2743034
A/chicken/Hiroshima/22F9T/2023|2023-01-21|NA|A / H5N1|EPI2751331
A/northern goshawk/Korea/22WC428/2023|2023-01-22|NA|A / H5N1|EPI2743154
A/Eurasian eagle owl/Korea/22WC032/2022|2022-11-15|NA|A / H5N1|EPI2742482
A/egret/Korea/22WC406/2023|2023-01-13|NA|A / H5N1|EPI2743106
A/chicken/Miyazaki/22A8T/2022|2022-11-20|NA|A / H5N1|EPI2747737
A/water/Tottori/NK1220-1/2022|2022-12-20|NA|A / H5N1|EPI3077293
A/Bean goose/Korea/22WC059/2022|2022-11-21|NA|A / H5N1|EPI2742530
A/hooded crane/Kagoshima/KU-7/2022 H5N1 |2022-11-04|NA|A / H5N1|EPI2788349
A/hooded crane/Kagoshima/KU-186/2022 H5N1 |2022-12-07|NA|A / H5N1|EPI2789293
A/environment/Kagoshima/KU-D3/2022 H5N1 |2022-11-06|NA|A / H5N1|EPI2789580
A/Hooded crane/Korea/22WC109/2022|2022-11-28|NA|A / H5N1|EPI2742698
A/hooded crane/Kagoshima/KU-189/2022 H5N1 |2022-12-08|NA|A / H5N1|EPI2789301
A/eagle/Korea/22WC464/2023|2023-01-06|NA|A / H5N1|EPI2743178
A/white-fronted goose/Korea/22WC116/2022|2022-11-28|NA|A / H5N1|EPI2742682
A/Spot-billed duck/Korea/K22-920/2022|2022-11-21|NA|A / H5N1|EPI2231781
A/pelican/Wakayama/3011003/2022|2022-11-13|NA|A / H5N1|EPI3599658
A/Em/Korea/22WF123-24P/2022|2022-11-02|NA|A / H5N1|EPI2742426
A/crow/Japan/TU-05/2022|2022-12-26|NA|A / H5N1|EPI2528171
A/chicken/Fukuoka/22D2C/2023|2023-03-02|NA|A / H5N1|EPI2751163
A/chicken/Kagoshima/22J5T/2022|2022-12-18|NA|A / H5N1|EPI2751763
A/duck/Miyagi/22B12T/2023|2023-01-28|NA|A / H5N1|EPI2752253
A/guinea fowl/Ibaraki/22D3T/2023|2023-02-02|NA|A / H5N1|EPI2752333
A/mallard/Shizuoka/2212D006/2022|2022-12-26|NA|A / H5N1|EPI2412728
A/whooper swan/Miyagi/0402B006/2023|2023-02-17|NA|A / H5N1|EPI2666990
A/Em/Korea/22WF182-9P/2022|2022-11-15|NA|A / H5N1|EPI2742514
A/duck/Chiba/22F4T/2023|2023-02-10|NA|A / H5N1|EPI2752245
A/black-headed gull/Korea/22WC117/2022|2022-11-28|NA|A / H5N1|EPI2742706
A/chicken/Wakayama/22B3T/2022|2022-11-30|NA|A / H5N1|EPI2752183
A/duck/Bangladesh/58825/2023|2023-09-09|NA|A / H5N1|EPI3226769
A/duck/Bangladesh/SJCEIRR-58401/2023|2023-06-23|NA|A / H5N1|EPI2893590
A/duck/Bangladesh/61900/2024|2024-02-23|NA|A / H5N1|EPI3559785
A/duck/Bangladesh/61908/2024|2024-02-23|NA|A / H5N1|EPI3559878
A/whooper swan/Korea/22WC040/2022|2022-11-17|NA|A / H5N1|EPI2742522
A/chicken/Okinawa/22A7T/2022|2022-12-16|NA|A / H5N1|EPI2752095
A/duck/Jiangxi/631/2024|2024-04-01|NA|A / H5N1|EPI3567866
A/chicken/Kagawa/22D9T/2022|2022-12-11|NA|A / H5N1|EPI2751611
A/chicken/Niigata/22D5T/2023|2023-03-06|NA|A / H5N1|EPI2751930
A/chicken/Ibaraki/22E1T/2023|2023-02-03|NA|A / H5N1|EPI2751515
A/white-fronted goose/Korea/22WC365/2023|2023-01-09|NA|A / H5N1|EPI2743018
A/white-fronted goose/Korea/22WC401-3P/2023|2023-01-13|NA|A / H5N1|EPI2743082
A/Eurasian eagle owl/Korea/22WC500/2023|2023-02-07|NA|A / H5N1|EPI2743234
A/duck/Aichi/22B4C/2022|2022-12-08|NA|A / H5N1|EPI2752223
A/chicken/Hokkaido/22E9C/2023|2023-04-07|NA|A / H5N1|EPI2751427
A/long-eared owl/Korea/22WC586/2023|2023-03-03|NA|A / H5N1|EPI2743266
A/white-naped crane/Korea/22WC366/2023|2023-01-09|NA|A / H5N1|EPI2743026
A/environment/Kagoshima/KU-J7/2022 H5N1 |2022-11-09|NA|A / H5N1|EPI2789500
A/white-fronted goose/Korea/22WC244/2022|2022-12-23|NA|A / H5N1|EPI2742850
A/Bean goose/Korea/22WC427/2023|2023-01-20|NA|A / H5N1|EPI2743146
A/little egret/Korea/22WC572/2023|2023-02-28|NA|A / H5N1|EPI2743258
A/chicken/Kagoshima/22M3T/2023|2023-02-03|NA|A / H5N1|EPI2751819
A/gray heron/Korea/22WC426/2023|2023-01-20|NA|A / H5N1|EPI2743138
A/egret/Korea/22WC281/2022|2022-12-27|NA|A / H5N1|EPI2742906
A/common buzzard/Korea/22WC336/2023|2023-01-05|NA|A / H5N1|EPI2742994
A/egret/Korea/22WC394/2023|2023-01-13|NA|A / H5N1|EPI2743090
A/duck/Vietnam/HU16-NS82/2023|2023-06-04|NA|A / H5N1|EPI2737497
A/chicken/Philippines/BA-MHN/2022|2022-07-08|NA|A / H5N1|EPI3221709
A/large-billed crow/Fukuoka/4002A005/2023|2023-02-06|NA|A / H5N1|EPI2667038
A/Eurasian Jay/Tochigi/0902A011/2023|2023-02-20|NA|A / H5N1|EPI2667022
A/eastern buzzard/Tochigi/0901B015/2023|2023-01-18|NA|A / H5N1|EPI2487059

MP

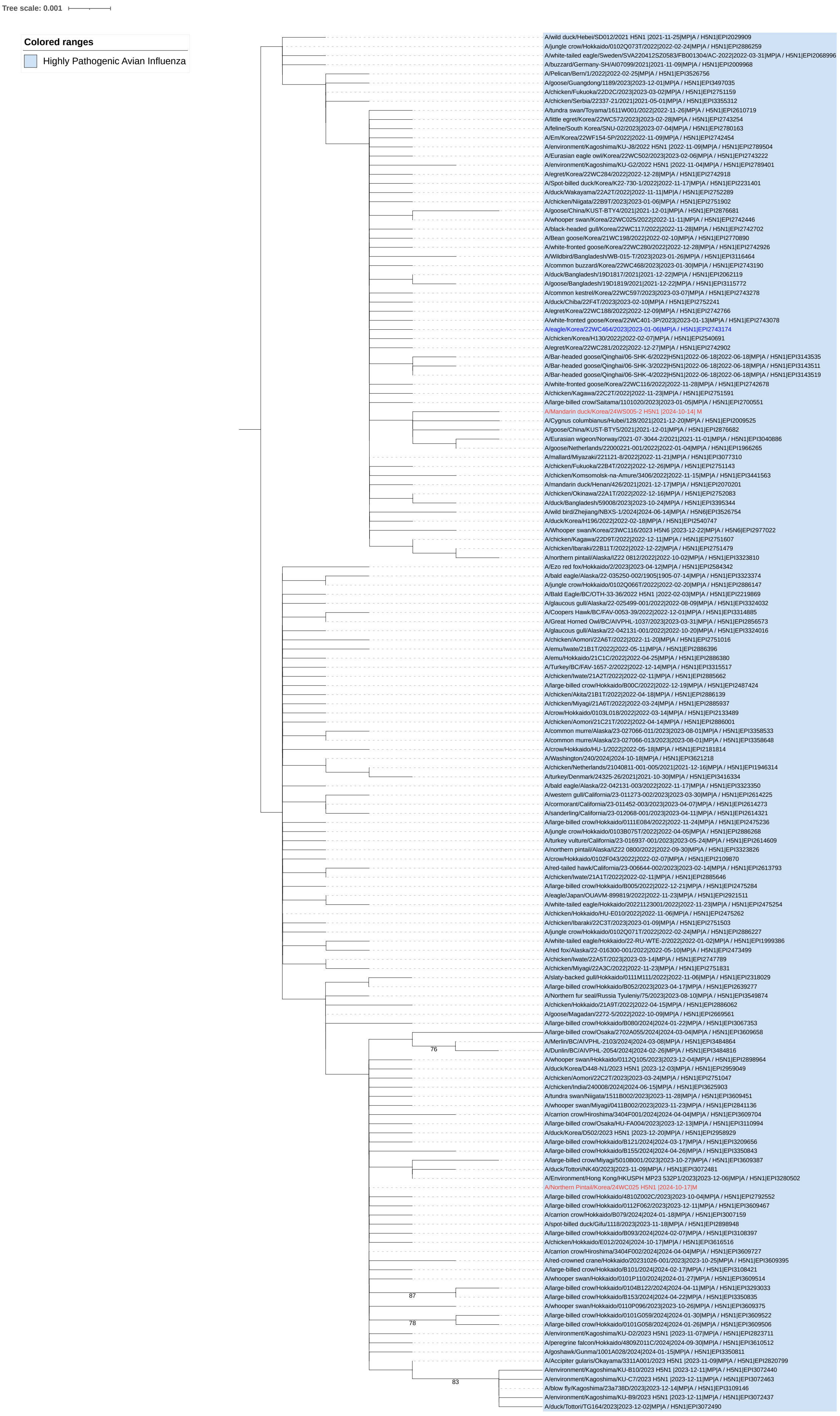

Tree scale: 0.001
Colored ranges
Highly Pathogenic Avian Influenza
A/wild duck/Hebei/SD012/2021 H5N1 |2021-11-25|MP|A / H5N1|EPI2029909
A/jungle crow/Hokkaido/0102Q073T/2022|2022-02-24|MP|A / H5N1|EPI2886259
A/white-tailed eagle/Sweden/SVA220412SZ0583/FB001304/AC-2022|2022-03-31|MP|A / H5N1|EPI2068996
A/buzzard/Germany-SH/AI07099/2021|2021-11-09|MP|A / H5N1|EPI2009968
A/Pelican/Bern/1/2022|2022-02-25|MP|A / H5N1|EPI3526756
A/goose/Guangdong/1189/2023|2023-12-01|MP|A / H5N1|EPI3497035
A/chicken/Fukuoka/22D2C/2023|2023-03-02|MP|A / H5N1|EPI2751159
A/chicken/Serbia/22337-21/2021|2021-05-01|MP|A / H5N1|EPI3355312
A/tundra swan/Toyama/1611W001/2022|2022-11-26|MP|A / H5N1|EPI2610719
A/little egret/Korea/22WC572/2023|2023-02-28|MP|A / H5N1|EPI2743254
A/feline/South Korea/SNU-02/2023|2023-07-04|MP|A / H5N1|EPI2780163
A/Em/Korea/22WF154-5P/2022|2022-11-09|MP|A / H5N1|EPI2742454
A/environment/Kagoshima/KU-J8/2022 H5N1 |2022-11-09|MP|A / H5N1|EPI2789504
A/Eurasian eagle owl/Korea/22WC502/2023|2023-02-06|MP|A / H5N1|EPI2743222
A/environment/Kagoshima/KU-G2/2022 H5N1 |2022-11-04|MP|A / H5N1|EPI2789401
A/egret/Korea/22WC284/2022|2022-12-28|MP|A / H5N1|EPI2742918
A/Spot-billed duck/Korea/K22-730-1/2022|2022-11-17|MP|A / H5N1|EPI2231401
A/duck/Wakayama/22A2T/2022|2022-11-11|MP|A / H5N1|EPI2752289
A/chicken/Niigata/22B9T/2023|2023-01-06|MP|A / H5N1|EPI2751902
A/goose/China/KUST-BTY4/2021|2021-12-01|MP|A / H5N1|EPI2876681
A/whooper swan/Korea/22WC025/2022|2022-11-11|MP|A / H5N1|EPI2742446
A/black-headed gull/Korea/22WC117/2022|2022-11-28|MP|A / H5N1|EPI2742702
A/Bean goose/Korea/21WC198/2022|2022-02-10|MP|A / H5N1|EPI2770890
A/white-fronted goose/Korea/22WC280/2022|2022-12-28|MP|A / H5N1|EPI2742926
A/Wildbird/Bangladesh/WB-015-T/2023|2023-01-26|MP|A / H5N1|EPI3116464
A/common buzzard/Korea/22WC468/2023|2023-01-30|MP|A / H5N1|EPI2743190
A/duck/Bangladesh/19D1817/2021|2021-12-22|MP|A / H5N1|EPI2062119
A/goose/Bangladesh/19D1819/2021|2021-12-22|MP|A / H5N1|EPI3115772
A/common kestrel/Korea/22WC597/2023|2023-03-07|MP|A / H5N1|EPI2743278
A/duck/Chiba/22F4T/2023|2023-02-10|MP|A / H5N1|EPI2752241
A/egret/Korea/22WC188/2022|2022-12-09|MP|A / H5N1|EPI2742766
A/white-fronted goose/Korea/22WC401-3P/2023|2023-01-13|MP|A / H5N1|EPI2743078
A/eagle/Korea/22WC464/2023|2023-01-06|MP|A / H5N1|EPI2743174
A/chicken/Korea/H130/2022|2022-02-07|MP|A / H5N1|EPI2540691
A/egret/Korea/22WC281/2022|2022-12-27|MP|A / H5N1|EPI2742902
A/Bar-headed goose/Qinghai/06-SHK-6/2022|H5N1|2022-06-18|MP|A / H5N1|EPI3143535
A/Bar-headed goose/Qinghai/06-SHK-3/2022|H5N1|2022-06-18|MP|A / H5N1|EPI3143511
A/Bar-headed goose/Qinghai/06-SHK-4/2022|H5N1|2022-06-18|MP|A / H5N1|EPI3143519
A/white-fronted goose/Korea/22WC116/2022|2022-11-28|MP|A / H5N1|EPI2742678
A/chicken/Kagawa/22C2T/2022|2022-11-23|MP|A / H5N1|EPI2751591
A/large-billed crow/Saitama/1101020/2023|2023-01-05|MP|A / H5N1|EPI2700551
A/Mandarin duck/Korea/24WS005-2 H5N1 |2024-10-14| M
A/Cygnus columbianus/Hubei/128/2021|2021-12-20|MP|A / H5N1|EPI2009525
A/goose/China/KUST-BTY5/2021|2021-12-01|MP|A / H5N1|EPI2876682
A/Eurasian wigeon/Norway/2021-07-3044-2/2021|2021-11-01|MP|A / H5N1|EPI3040886
A/goose/Netherlands/22000221-001/2022|2022-01-04|MP|A / H5N1|EPI1966265
A/mallard/Miyazaki/221121-8/2022|2022-11-21|MP|A / H5N1|EPI3077310
A/chicken/Fukuoka/22B4T/2022|2022-12-26|MP|A / H5N1|EPI2751143
A/chicken/Komsomolsk-na-Amure/3406/2022|2022-11-15|MP|A / H5N1|EPI3441563
A/mandarin duck/Henan/426/2021|2021-12-17|MP|A / H5N1|EPI2070201
A/chicken/Okinawa/22A1T/2022|2022-12-16|MP|A / H5N1|EPI2752083
A/duck/Bangladesh/59008/2023|2023-10-24|MP|A / H5N1|EPI3395344
A/wild bird/Zhejiang/NBXS-1/2024|2024-06-14|MP|A / H5N6|EPI3526754
A/duck/Korea/H196/2022|2022-02-18|MP|A / H5N1|EPI2540747
A/Whooper swan/Korea/23WC116/2023 H5N6 |2023-12-22|MP|A / H5N6|EPI2977022
A/chicken/Kagawa/22D9T/2022|2022-12-11|MP|A / H5N1|EPI2751607
A/chicken/Ibaraki/22B11T/2022|2022-12-22|MP|A / H5N1|EPI2751479
A/northern pintail/Alaska/IZ22 0812/2022|2022-10-02|MP|A / H5N1|EPI3323810
A/Ezo red fox/Hokkaido/2/2023|2023-04-12|MP|A / H5N1|EPI2584342
A/bald eagle/Alaska/22-035250-002/1905|1905-07-14|MP|A / H5N1|EPI3323374
A/jungle crow/Hokkaido/0102Q066T/2022|2022-02-20|MP|A / H5N1|EPI2886147
A/Bald Eagle/BC/OTH-33-36/2022 H5N1 |2022-02-03|MP|A / H5N1|EPI2219869
A/glaucous gull/Alaska/22-025499-001/2022|2022-08-09|MP|A / H5N1|EPI3324032
A/Coopers Hawk/BC/FAV-0053-39/2022|2022-12-01|MP|A / H5N1|EPI3314885
A/Great Horned Owl/BC/AIVPHL-1037/2023|2023-03-31|MP|A / H5N1|EPI2856573
A/glaucous gull/Alaska/22-042131-001/2022|2022-10-20|MP|A / H5N1|EPI3324016
A/chicken/Aomori/22A6T/2022|2022-11-20|MP|A / H5N1|EPI2751016
A/emu/Iwate/21B1T/2022|2022-05-11|MP|A / H5N1|EPI2886396
A/emu/Hokkaido/21C1C/2022|2022-04-25|MP|A / H5N1|EPI2886380
A/Turkey/BC/FAV-1657-2/2022|2022-12-14|MP|A / H5N1|EPI3315517
A/chicken/Iwate/21A2T/2022|2022-02-11|MP|A / H5N1|EPI2885662
A/large-billed crow/Hokkaido/B00C/2022|2022-12-19|MP|A / H5N1|EPI2487424
A/chicken/Akita/21B1T/2022|2022-04-18|MP|A / H5N1|EPI2886139
A/chicken/Miyagi/21A6T/2022|2022-03-24|MP|A / H5N1|EPI2885937
A/crow/Hokkaido/0103L018/2022|2022-03-14|MP|A / H5N1|EPI2133489
A/chicken/Aomori/21C21T/2022|2022-04-14|MP|A / H5N1|EPI2886001
A/common murre/Alaska/23-027066-011/2023|2023-08-01|MP|A / H5N1|EPI3358533
A/common murre/Alaska/23-027066-013/2023|2023-08-01|MP|A / H5N1|EPI3358648
A/crow/Hokkaido/HU-1/2022|2022-05-18|MP|A / H5N1|EPI2181814
A/Washington/240/2024|2024-10-18|MP|A / H5N1|EPI3621218
A/chicken/Netherlands/21040811-001-005/2021|2021-12-16|MP|A / H5N1|EPI1946314
A/turkey/Denmark/24325-26/2021|2021-10-30|MP|A / H5N1|EPI3416334
A/bald eagle/Alaska/22-042131-003/2022|2022-11-17|MP|A / H5N1|EPI3323350
A/western gull/California/23-011273-002/2023|2023-03-30|MP|A / H5N1|EPI2614225
A/cormorant/California/23-011452-003/2023|2023-04-07|MP|A / H5N1|EPI2614273
A/sanderling/California/23-012068-001/2023|2023-04-11|MP|A / H5N1|EPI2614321
A/large-billed crow/Hokkaido/0111E084/2022|2022-11-24|MP|A / H5N1|EPI2475236
A/jungle crow/Hokkaido/0103B075T/2022|2022-04-05|MP|A / H5N1|EPI2886268
A/turkey vulture/California/23-016937-001/2023|2023-05-24|MP|A / H5N1|EPI2614609
A/northern pintail/Alaska/IZ22 0800/2022|2022-09-30|MP|A / H5N1|EPI3323826
A/crow/Hokkaido/0102F043/2022|2022-02-07|MP|A / H5N1|EPI2109870
A/red-tailed hawk/California/23-006644-002/2023|2023-02-14|MP|A / H5N1|EPI2613793
A/chicken/Iwate/21A1T/2022|2022-02-11|MP|A / H5N1|EPI2885646
A/large-billed crow/Hokkaido/B005/2022|2022-12-21|MP|A / H5N1|EPI2475284
A/eagle/Japan/OUAVM-899819/2022|2022-11-23|MP|A / H5N1|EPI2921511
A/white-tailed eagle/Hokkaido/20221123001/2022|2022-11-23|MP|A / H5N1|EPI2475254
A/chicken/Hokkaido/HU-E010/2022|2022-11-06|MP|A / H5N1|EPI2475262
A/chicken/Ibaraki/22C3T/2023|2023-01-09|MP|A / H5N1|EPI2751503
A/jungle crow/Hokkaido/0102Q071T/2022|2022-02-24|MP|A / H5N1|EPI2886227
A/white-tailed eagle/Hokkaido/22-RU-WTE-2/2022|2022-01-02|MP|A / H5N1|EPI1999386
A/red fox/Alaska/22-016300-001/2022|2022-05-10|MP|A / H5N1|EPI2473499
A/chicken/Iwate/22A5T/2023|2023-03-14|MP|A / H5N1|EPI2747789
A/chicken/Miyagi/22A3C/2022|2022-11-23|MP|A / H5N1|EPI2751831
A/slaty-backed gull/Hokkaido/0111M111/2022|2022-11-06|MP|A / H5N1|EPI2318029
A/large-billed crow/Hokkaido/B052/2023|2023-04-17|MP|A / H5N1|EPI2639277
A/Northern fur seal/Russia Tyuleniy/75/2023|2023-08-10|MP|A / H5N1|EPI3549874
A/chicken/Hokkaido/21A9T/2022|2022-04-15|MP|A / H5N1|EPI2886062
A/goose/Magadan/2272-5/2022|2022-10-09|MP|A / H5N1|EPI2669561
A/large-billed crow/Hokkaido/B080/2024|2024-01-22|MP|A / H5N1|EPI3067353
A/large-billed crow/Osaka/2702A055/2024|2024-03-04|MP|A / H5N1|EPI3609658
A/Merlin/BC/AIVPHL-2103/2024|2024-03-08|MP|A / H5N1|EPI3484864
76
A/Dunlin/BC/AIVPHL-2054/2024|2024-02-26|MP|A / H5N1|EPI3484816
A/whooper swan/Hokkaido/0112Q105/2023|2023-12-04|MP|A / H5N1|EPI2898964
A/duck/Korea/D448-N1/2023 H5N1 |2023-12-03|MP|A / H5N1|EPI2959049
A/chicken/Aomori/22C2T/2023|2023-03-24|MP|A / H5N1|EPI2751047
A/chicken/India/240008/2024|2024-06-15|MP|A / H5N1|EPI3625903
A/tundra swan/Niigata/1511B002/2023|2023-11-28|MP|A / H5N1|EPI3609451
A/whooper swan/Miyagi/0411B002/2023|2023-11-23|MP|A / H5N1|EPI2841136
A/carrion crow/Hiroshima/3404F001/2024|2024-04-04|MP|A / H5N1|EPI3609704
A/large-billed crow/Osaka/HU-FA004/2023|2023-12-13|MP|A / H5N1|EPI3110994
A/duck/Korea/D502/2023 H5N1 |2023-12-20|MP|A / H5N1|EPI2958929
A/large-billed crow/Hokkaido/B121/2024|2024-03-17|MP|A / H5N1|EPI3209656
A/large-billed crow/Hokkaido/B155/2024|2024-04-26|MP|A / H5N1|EPI3350843
A/large-billed crow/Miyagi/5010B001/2023|2023-10-27|MP|A / H5N1|EPI3609387
A/duck/Tottori/NK40/2023|2023-11-09|MP|A / H5N1|EPI3072481
A/Environment/Hong Kong/HKUSPH MP23 532P1/2023|2023-12-06|MP|A / H5N1|EPI3280502
A/Northern Pintail/Korea/24WC025 H5N1 |2024-10-17|M
A/large-billed crow/Hokkaido/4810Z002C/2023|2023-10-04|MP|A / H5N1|EPI2792552
A/large-billed crow/Hokkaido/0112F062/2023|2023-12-11|MP|A / H5N1|EPI3609467
A/carrion crow/Hokkaido/B079/2024|2024-01-18|MP|A / H5N1|EPI3007159
A/spot-billed duck/Gifu/1118/2023|2023-11-18|MP|A / H5N1|EPI2898948
A/large-billed crow/Hokkaido/B093/2024|2024-02-07|MP|A / H5N1|EPI3108397
A/chicken/Hokkaido/E012/2024|2024-10-17|MP|A / H5N1|EPI3616516
A/carrion crow/Hiroshima/3404F002/2024|2024-04-04|MP|A / H5N1|EPI3609727
A/red-crowned crane/Hokkaido/20231026-001/2023|2023-10-25|MP|A / H5N1|EPI3609395
A/large-billed crow/Hokkaido/B101/2024|2024-02-17|MP|A / H5N1|EPI3108421
A/whooper swan/Hokkaido/0101P110/2024|2024-01-27|MP|A / H5N1|EPI3609514
A/large-billed crow/Hokkaido/0104B122/2024|2024-04-11|MP|A / H5N1|EPI3293033
87
A/large-billed crow/Hokkaido/B153/2024|2024-04-22|MP|A / H5N1|EPI3350835
A/whooper swan/Hokkaido/0110P096/2023|2023-10-26|MP|A / H5N1|EPI3609375
A/large-billed crow/Hokkaido/0101G059/2024|2024-01-30|MP|A / H5N1|EPI3609522
78
A/large-billed crow/Hokkaido/0101G058/2024|2024-01-26|MP|A / H5N1|EPI3609506
A/environment/Kagoshima/KU-D2/2023 H5N1 |2023-11-07|MP|A / H5N1|EPI2823711
A/peregrine falcon/Hokkaido/4809Z011C/2024|2024-09-30|MP|A / H5N1|EPI3610512
A/goshawk/Gunma/1001A028/2024|2024-01-15|MP|A / H5N1|EPI3350811
A/Accipiter gularis/Okayama/3311A001/2023 H5N1 |2023-11-09|MP|A / H5N1|EPI2820799
A/environment/Kagoshima/KU-B10/2023 H5N1 |2023-12-11|MP|A / H5N1|EPI3072440
83
A/environment/Kagoshima/KU-C7/2023 H5N1 |2023-12-11|MP|A / H5N1|EPI3072463
A/blow fly/Kagoshima/23a738D/2023|2023-12-14|MP|A / H5N1|EPI3109146
A/environment/Kagoshima/KU-B9/2023 H5N1 |2023-12-11|MP|A / H5N1|EPI3072437
A/duck/Tottori/TG164/2023|2023-12-02|MP|A / H5N1|EPI3072490

NS

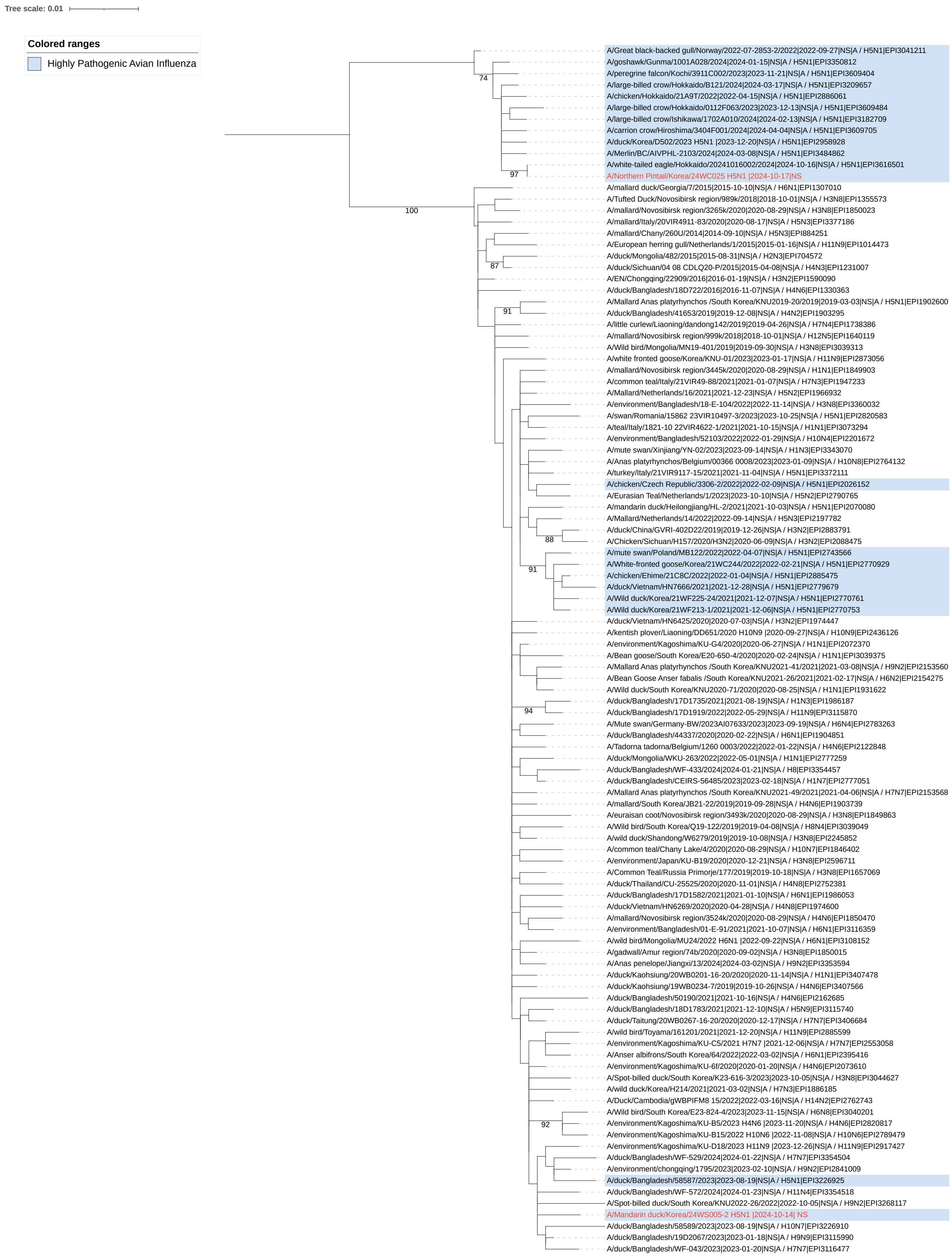

Tree scale: 0.01
Colored ranges
Highly Pathogenic Avian Influenza
74
97
100
87
91
91
88
91
94
92
A/Great black-backed gull/Norway/2022-07-2853-2/2022|2022-09-27|NS|A / H5N1|EPI3041211
A/goshawk/Gunma/1001A028/2024|2024-01-15|NS|A / H5N1|EPI3350812
A/peregrine falcon/Kochi/3911C002/2023|2023-11-21|NS|A / H5N1|EPI3609404
A/large-billed crow/Hokkaido/B121/2024|2024-03-17|NS|A / H5N1|EPI3209657
A/chicken/Hokkaido/21A9T/2022|2022-04-15|NS|A / H5N1|EPI2886061
A/large-billed crow/Hokkaido/0112F063/2023|2023-12-13|NS|A / H5N1|EPI3609484
A/large-billed crow/Ishikawa/1702A010/2024|2024-02-13|NS|A / H5N1|EPI3182709
A/carrion crow/Hiroshima/3404F001/2024|2024-04-04|NS|A / H5N1|EPI3609705
A/duck/Korea/D502/2023 H5N1 |2023-12-20|NS|A / H5N1|EPI2958928
A/Merlin/BC/AIVPHL-2103/2024|2024-03-08|NS|A / H5N1|EPI3484862
A/white-tailed eagle/Hokkaido/20241016002/2024|2024-10-16|NS|A / H5N1|EPI3616501
A/Northern Pintail/Korea/24WC025 H5N1 |2024-10-17|NS
A/mallard duck/Georgia/7/2015|2015-10-10|NS|A / H6N1|EPI1307010
A/Tufted Duck/Novosibirsk region/989k/2018|2018-10-01|NS|A / H3N8|EPI1355573
A/mallard/Novosibirsk region/3265k/2020|2020-08-29|NS|A / H3N8|EPI1850023
A/mallard/Italy/20VIR4911-83/2020|2020-08-17|NS|A / H5N3|EPI3377186
A/mallard/Chany/260U/2014|2014-09-10|NS|A / H5N3|EPI884251
A/European herring gull/Netherlands/1/2015|2015-01-16|NS|A / H11N9|EPI1014473
A/duck/Mongolia/482/2015|2015-08-31|NS|A / H2N3|EPI704572
A/duck/Sichuan/04 08 CDLQ20-P/2015|2015-04-08|NS|A / H4N3|EPI1231007
A/EN/Chongqing/22909/2016|2016-01-19|NS|A / H3N2|EPI1590090
A/duck/Bangladesh/18D722/2016|2016-11-07|NS|A / H4N6|EPI1330363
A/Mallard Anas platyrhynchos /South Korea/KNU2019-20/2019|2019-03-03|NS|A / H5N1|EPI1902600
A/duck/Bangladesh/41653/2019|2019-12-08|NS|A / H4N2|EPI1903295
A/little curlew/Liaoning/dandong142/2019|2019-04-26|NS|A / H7N4|EPI1738386
A/mallard/Novosibirsk region/999k/2018|2018-10-01|NS|A / H12N5|EPI1640119
A/Wild bird/Mongolia/MN19-401/2019|2019-09-30|NS|A / H3N8|EPI3039313
A/white fronted goose/Korea/KNU-01/2023|2023-01-17|NS|A / H11N9|EPI2873056
A/mallard/Novosibirsk region/3445k/2020|2020-08-29|NS|A / H1N1|EPI1849903
A/common teal/Italy/21VIR49-88/2021|2021-01-07|NS|A / H7N3|EPI1947233
A/Mallard/Netherlands/16/2021|2021-12-23|NS|A / H5N2|EPI1966932
A/environment/Bangladesh/18-E-104/2022|2022-11-14|NS|A / H3N8|EPI3360032
A/swan/Romania/15862 23VIR10497-3/2023|2023-10-25|NS|A / H5N1|EPI2820583
A/teal/Italy/1821-10 22VIR4622-1/2021|2021-10-15|NS|A / H1N1|EPI3073294
A/environment/Bangladesh/52103/2022|2022-01-29|NS|A / H10N4|EPI2201672
A/mute swan/Xinjiang/YN-02/2023|2023-09-14|NS|A / H1N3|EPI3343070
A/Anas platyrhynchos/Belgium/00366 0008/2023|2023-01-09|NS|A / H10N8|EPI2764132
A/turkey/Italy/21VIR9117-15/2021|2021-11-04|NS|A / H5N1|EPI3372111
A/chicken/Czech Republic/3306-2/2022|2022-02-09|NS|A / H5N1|EPI2026152
A/Eurasian Teal/Netherlands/1/2023|2023-10-10|NS|A / H5N2|EPI2790765
A/mandarin duck/Heilongjiang/HL-2/2021|2021-10-03|NS|A / H5N1|EPI2070080
A/Mallard/Netherlands/14/2022|2022-09-14|NS|A / H5N3|EPI2197782
A/duck/China/GVRI-402D22/2019|2019-12-26|NS|A / H3N2|EPI2883791
A/Chicken/Sichuan/H157/2020/H3N2|2020-06-09|NS|A / H3N2|EPI2088475
A/mute swan/Poland/MB122/2022|2022-04-07|NS|A / H5N1|EPI2743566
A/White-fronted goose/Korea/21WC244/2022|2022-02-21|NS|A / H5N1|EPI2770929
A/chicken/Ehime/21C8C/2022|2022-01-04|NS|A / H5N1|EPI2885475
A/duck/Vietnam/HN7666/2021|2021-12-28|NS|A / H5N1|EPI2779679
A/Wild duck/Korea/21WF225-24/2021|2021-12-07|NS|A / H5N1|EPI2770761
A/Wild duck/Korea/21WF213-1/2021|2021-12-06|NS|A / H5N1|EPI2770753
A/duck/Vietnam/HN6425/2020|2020-07-03|NS|A / H3N2|EPI1974447
A/kentish plover/Liaoning/DD651/2020 H10N9 |2020-09-27|NS|A / H10N9|EPI2436126
A/environment/Kagoshima/KU-G4/2020|2020-06-27|NS|A / H1N1|EPI2072370
A/Bean goose/South Korea/E20-650-4/2020|2020-02-24|NS|A / H1N1|EPI3039375
A/Mallard Anas platyrhynchos /South Korea/KNU2021-41/2021|2021-03-08|NS|A / H9N2|EPI2153560
A/Bean Goose Anser fabalis /South Korea/KNU2021-26/2021|2021-02-17|NS|A / H6N2|EPI2154275
A/Wild duck/South Korea/KNU2020-71/2020|2020-08-25|NS|A / H1N1|EPI1931622
A/duck/Bangladesh/17D1735/2021|2021-08-19|NS|A / H1N3|EPI1986187
A/duck/Bangladesh/17D1919/2022|2022-05-29|NS|A / H11N9|EPI3115870
A/Mute swan/Germany-BW/2023AI07633/2023|2023-09-19|NS|A / H6N4|EPI2783263
A/duck/Bangladesh/44337/2020|2020-02-22|NS|A / H6N1|EPI1904851
A/Tadorna tadorna/Belgium/1260 0003/2022|2022-01-22|NS|A / H4N6|EPI2122848
A/duck/Mongolia/WKU-263/2022|2022-05-01|NS|A / H1N1|EPI2777259
A/duck/Bangladesh/WF-433/2024|2024-01-21|NS|A / H8|EPI3354457
A/duck/Bangladesh/CEIRS-56485/2023|2023-02-18|NS|A / H1N7|EPI2777051
A/Mallard Anas platyrhynchos /South Korea/KNU2021-49/2021|2021-04-06|NS|A / H7N7|EPI2153568
A/mallard/South Korea/JB21-22/2019|2019-09-28|NS|A / H4N6|EPI1903739
A/euraisan coot/Novosibirsk region/3493k/2020|2020-08-29|NS|A / H3N8|EPI1849863
A/Wild bird/South Korea/Q19-122/2019|2019-04-08|NS|A / H8N4|EPI3039049
A/wild duck/Shandong/W6279/2019|2019-10-08|NS|A / H3N8|EPI2245852
A/common teal/Chany Lake/4/2020|2020-08-29|NS|A / H10N7|EPI1846402
A/environment/Japan/KU-B19/2020|2020-12-21|NS|A / H3N8|EPI2596711
A/Common Teal/Russia Primorje/177/2019|2019-10-18|NS|A / H3N8|EPI1657069
A/duck/Thailand/CU-25525/2020|2020-11-01|NS|A / H4N8|EPI2752381
A/duck/Bangladesh/17D1582/2021|2021-01-10|NS|A / H6N1|EPI1986053
A/duck/Vietnam/HN6269/2020|2020-04-28|NS|A / H4N8|EPI1974600
A/mallard/Novosibirsk region/3524k/2020|2020-08-29|NS|A / H4N6|EPI1850470
A/environment/Bangladesh/01-E-91/2021|2021-10-07|NS|A / H6N1|EPI3116359
A/wild bird/Mongolia/MU24/2022 H6N1 |2022-09-22|NS|A / H6N1|EPI3108152
A/gadwall/Amur region/74b/2020|2020-09-02|NS|A / H3N8|EPI1850015
A/Anas penelope/Jiangxi/13/2024|2024-03-02|NS|A / H9N2|EPI3353594
A/duck/Kaohsiung/20WB0201-16-20/2020|2020-11-14|NS|A / H1N1|EPI3407478
A/duck/Kaohsiung/19WB0234-7/2019|2019-10-26|NS|A / H4N6|EPI3407566
A/duck/Bangladesh/50190/2021|2021-10-16|NS|A / H4N6|EPI2162685
A/duck/Bangladesh/18D1783/2021|2021-12-10|NS|A / H5N9|EPI3115740
A/duck/Taitung/20WB0267-16-20/2020|2020-12-17|NS|A / H7N7|EPI3406684
A/wild bird/Toyama/161201/2021|2021-12-20|NS|A / H11N9|EPI2885599
A/environment/Kagoshima/KU-C5/2021 H7N7 |2021-12-06|NS|A / H7N7|EPI2553058
A/Anser albifrons/South Korea/64/2022|2022-03-02|NS|A / H6N1|EPI2395416
A/environment/Kagoshima/KU-6f/2020|2020-01-20|NS|A / H4N6|EPI2073610
A/Spot-billed duck/South Korea/K23-616-3/2023|2023-10-05|NS|A / H3N8|EPI3044627
A/wild duck/Korea/H214/2021|2021-03-02|NS|A / H7N3|EPI1886185
A/Duck/Cambodia/gWBPIFM8 15/2022|2022-03-16|NS|A / H14N2|EPI2762743
A/Wild bird/South Korea/E23-824-4/2023|2023-11-15|NS|A / H6N8|EPI3040201
A/environment/Kagoshima/KU-B5/2023 H4N6 |2023-11-20|NS|A / H4N6|EPI2820817
A/environment/Kagoshima/KU-B15/2022 H10N6 |2022-11-08|NS|A / H10N6|EPI2789479
A/environment/Kagoshima/KU-D18/2023 H11N9 |2023-12-26|NS|A / H11N9|EPI2917427
A/duck/Bangladesh/WF-529/2024|2024-01-22|NS|A / H7N7|EPI3354504
A/environment/chongqing/1795/2023|2023-02-10|NS|A / H9N2|EPI2841009
A/duck/Bangladesh/58587/2023|2023-08-19|NS|A / H5N1|EPI3226925
A/duck/Bangladesh/WF-572/2024|2024-01-23|NS|A / H11N4|EPI3354518
A/Spot-billed duck/South Korea/KNU2022-26/2022|2022-10-05|NS|A / H9N2|EPI3268117
A/Mandarin duck/Korea/24WS005-2 H5N1 |2024-10-14| NS
A/duck/Bangladesh/58589/2023|2023-08-19|NS|A / H10N7|EPI3226910
A/duck/Bangladesh/19D2067/2023|2023-01-18|NS|A / H9N9|EPI3115990
A/duck/Bangladesh/WF-043/2023|2023-01-20|NS|A / H7N7|EPI3116477
