# Supplementary figures and images for: New incursions of H5N1 clade 2.3.4.4b highly pathogenic avian influenza viruses in wild birds, South Korea, October 2024
Source: Front Vet Sci. 2025 Jan 10;11:1526118. doi: 10.3389/fvets.2024.1526118 (PMC11758627; doi:10.3389/fvets.2024.1526118)

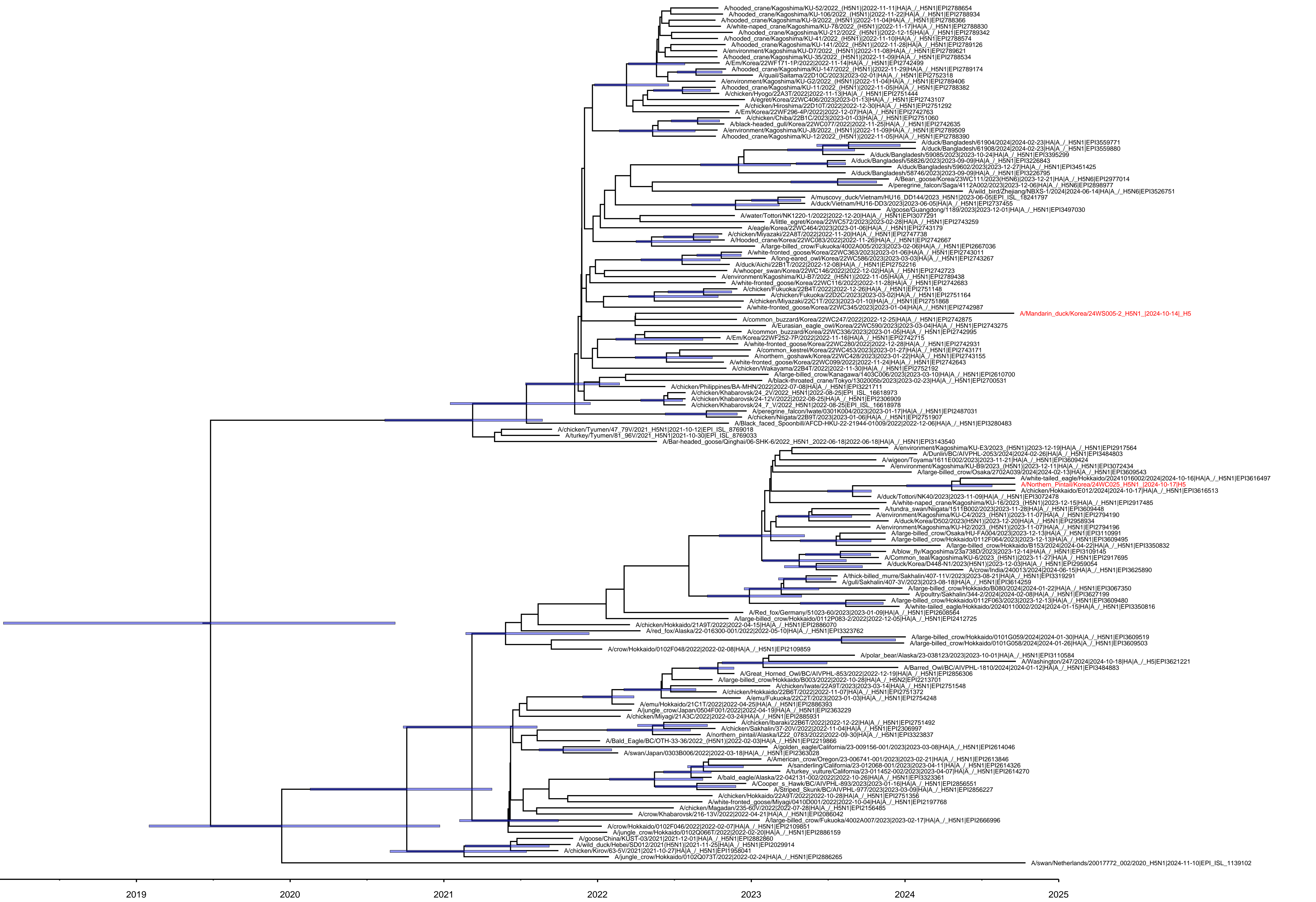

Supplement: Supplementary Figure 3 — Time-scaled Maximum clade credibility tree constructed using the hemagglutinin gene of clade 2.3.4.4 b H5N1 HPAI viruses. Red indicates H5N1 isolates from South Korea, October 2024. Node bars represent 95% HPD of the node height with a posterior probability >0.5. The horizontal axis represents the decimal year. [file Data_Sheet_3.pdf]
